# Widespread impact of immunoglobulin V-gene allelic polymorphisms on antibody reactivity

**DOI:** 10.1016/j.celrep.2023.113194

**Published:** 2023-09-30

**Authors:** Meng Yuan, Ziqi Feng, Huibin Lv, Natalie So, Ivana R. Shen, Timothy J.C. Tan, Qi Wen Teo, Wenhao O. Ouyang, Logan Talmage, Ian A. Wilson, Nicholas C. Wu

**Affiliations:** 1 Department of Integrative Structural and Computational Biology, The Scripps Research Institute, La Jolla, CA 92037, USA; 2 Department of Biochemistry, University of Illinois at Urbana-Champaign, Urbana, IL 61801, USA; 3 Carl R. Woese Institute for Genomic Biology, University of Illinois at Urbana-Champaign, Urbana, IL 61801, USA; 4 Department of Computer Science, University of Illinois at Urbana-Champaign, Urbana, IL 61801, USA; 5 Center for Biophysics and Quantitative Biology, University of Illinois at Urbana-Champaign, Urbana, IL 61801, USA; 6 The Skaggs Institute for Chemical Biology, The Scripps Research Institute, La Jolla, CA 92037, USA; 7 Carle Illinois College of Medicine, University of Illinois at Urbana-Champaign, Urbana, IL 61801, USA

**Keywords:** antibody, B cells, immunoglobulin V gene, allelic polymorphism, structural biology, paratope, virus

## Abstract

The ability of the human immune system to generate antibodies to any given antigen can be strongly influenced by immunoglobulin V-gene allelic polymorphisms. However, previous studies have provided only limited examples. Therefore, the prevalence of this phenomenon has been unclear. By analyzing >1,000 publicly available antibody-antigen structures, we show that many V-gene allelic polymorphisms in antibody paratopes are determinants for antibody binding activity. Biolayer interferometry experiments further demonstrate that paratope allelic polymorphisms on both heavy and light chains often abolish antibody binding. We also illustrate the importance of minor V-gene allelic polymorphisms with low frequency in several broadly neutralizing antibodies to severe acute respiratory syndrome coronavirus 2 (SARS-CoV-2) and influenza virus. Overall, this study not only highlights the pervasive impact of V-gene allelic polymorphisms on antibody binding but also provides mechanistic insights into the variability of antibody repertoires across individuals, which in turn have important implications for vaccine development and antibody discovery.

## Highlights

• Analysis of V-gene allelic polymorphisms in 1,048 antibody-antigen complex structures
• Many antibodies contain allelic polymorphisms predicted to disrupt binding
• These include antibodies with different V genes and to different pathogens
• Some broadly neutralizing antibodies depend on minor V-gene allelic polymorphisms

## Graphical abstract

**Figure:**
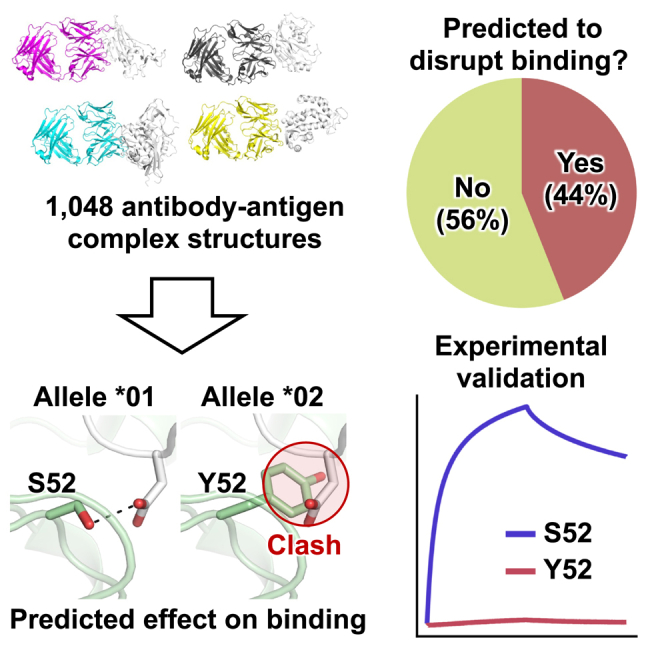

## Teaser

By analyzing >1,000 publicly available antibody-antigen complex structures, Yuan et al. demonstrate that antibody binding activity is often influenced by V-gene allelic polymorphisms. This result provides mechanistic insights into the variability of antibody repertoires across individuals, which in turn have important implications for vaccine development and antibody discovery.

## Introduction

Human antibodies, which are produced by B cells and composed of two chains (heavy and light), are central to the immune response against pathogen infection. To be able to recognize many different pathogens, the human antibody repertoire has enormous diversity, with up to 10^15^ unique antibody clonotypes.^1^ This diversity is generated mainly by V(D)J recombination, which is a somatic recombination process that assembles different germline gene segments, known as variable (V), diversity (D), and joining (J) genes, into the variable region of the antibody molecule. In addition, many V-gene segments are known to have multiple alleles that encode amino acid differences, which further increase the diversity of human antibody repertoire at the population level.

Allelic polymorphisms of immunoglobulin V genes (referred to as “V genes” hereafter) are enriched in the complementarity-determining regions (CDRs),^2^ which usually form the antibody paratopes (i.e., regions that involve in binding to antigens). Of note, allelic polymorphisms in this study refer to polymorphisms at the amino acid sequence level for convenience. Several studies have reported the importance of V-gene allelic polymorphisms in antibody binding. For example, allelic polymorphisms at IGHV1-69 residues 50 (G/R) and 54 (F/L) (n.b. Kabat numbering used throughout for all antibody residues) can influence antibody binding to severe acute respiratory syndrome coronavirus 2 (SARS-CoV-2).^3^ Allelic polymorphisms at IGHV1-69 residues 50 (G/R) can also impact antibody binding to *Staphylococcus aureus* (*S. aureus*).^4^ Other examples include IGHV3-33 residue 52 (W/S) in antibodies to *Plasmodium falciparum* (*P. falciparum*)^5^ and IGHV2-5 residue 54 (D/N) in antibodies to SARS-CoV-2^6^ and human immunodeficiency virus (HIV).^7^ Therefore, it is apparent that V-gene allelic polymorphisms can affect antibody binding. However, it remains unclear whether the impact of V-gene allelic polymorphisms on antibody binding is prevalent because previous studies typically characterized a single antibody-antigen pair at a time and the number of such studies is rather limited. Furthermore, previous studies of V-gene allelic polymorphisms mainly focused on the heavy chain,^3^^,^^4^^,^^5^^,^^6^^,^^7^^,^^8^^,^^9^^,^^10^ whereas those within the light chain remain largely unexplored.

Nevertheless, V-gene allelic polymorphisms are shown to have important public health relevance. For instance, IGHV1-2 allelic usage correlates with the response rate to an HIV vaccine candidate in a phase 1 clinical trial (ClinicalTrials.gov: NCT03547245),^8^^,^^9^^,^^11^ due to the influence of its allelic polymorphisms at residue 50 (W/R) on antibody binding to HIV.^8^^,^^12^^,^^13^ A previous study has also shown that IGHV1-69 allelic usage correlates with the broadly neutralizing antibody response to influenza virus in a vaccine cohort.^14^ This observation has also been attributed to the differential autoreactive propensities of different IGHV1-69 alleles,^10^ as well as a potentially minor effect of its allelic polymorphisms at residue 54 (L/F) on antibody binding to the conserved stem domain of influenza hemagglutinin (HA).^15^^,^^16^ Similarly, allele usages of IGHV3-66 and IGHV4-61 were associated with Kawasaki disease^17^ and rheumatic heart disease,^18^ respectively, although the underlying mechanisms are unknown. As a result, investigating the impact of V-gene allelic polymorphisms can provide critical insights into vaccine development and autoimmune diseases.^19^

In this study, we systematically investigate the effect of V-gene allelic polymorphisms on antibody binding by analyzing 1,048 publicly available antibody-antigen complex structures. V-gene allelic polymorphisms could be identified in the antibody paratope of 52% (544/1,048) complex structures. Computational analysis of protein mutational stability predicted that 73% of paratope allelic polymorphisms (i.e., mutating a paratope residue to alternative allelic polymorphisms) would disrupt antibody binding activity. The wide impact of V-gene allelic polymorphisms on antibody binding was further validated using biolayer interferometry (BLI). Our results also illustrated the importance of light-chain V-gene allelic polymorphisms. In addition, we identified several V-gene allelic polymorphisms that are essential for the binding activity of broadly neutralizing antibodies to SARS-CoV-2 and influenza virus yet have low frequency among antibodies in GenBank. These discoveries suggested that the importance of allelic polymorphisms on antibody reactivity may have been previously underestimated.

## Results

### Predicting V-gene allelic polymorphisms that weaken antibody binding activity

**Figure 1:**
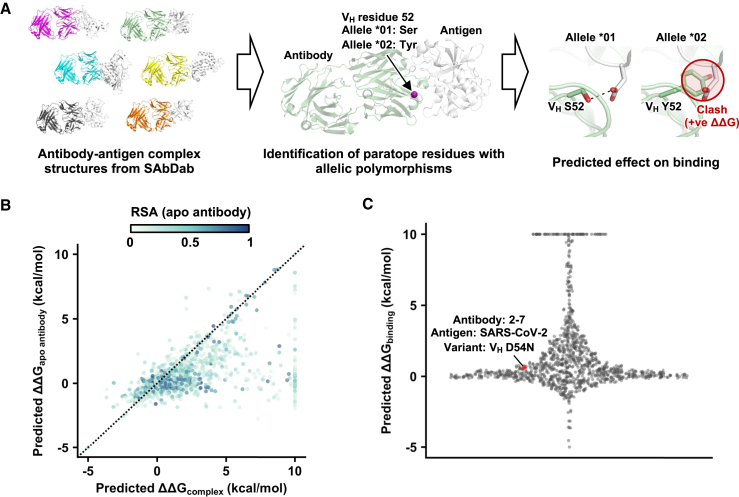
Predicting the effects of immunoglobulin V-gene allelic polymorphisms on antibody binding activity (A) Schematic of the analysis workflow (see STAR Methods). Briefly, human antibody-antigen complex structures were downloaded as PDB files from the Structural Antibody Database (SAbDab; http://opig.stats.ox.ac.uk/webapps/sabdab).^20^ Among these antibodies, paratope residues with allelic polymorphisms were identified. The effects of paratope allelic polymorphisms on antibody binding activity were then predicted using FoldX.^21^ (B) The relationship between predicted ΔΔG_apo antibody_ and predicted ΔΔG_complex_ is shown. Each data point is colored by the relative solvent accessibility (RSA) in the apo antibody. Residues that are fully solvent exposed have an RSA of 1, whereas those that are fully buried have an RSA of 0. (C) The distribution of predicted ΔΔG_binding_ of 1,150 paratope allelic polymorphisms of 544 antibody-antigen complex structures is shown. Predicted ΔΔG_binding_ was computed by predicted ΔΔG_complex_ – predicted ΔΔG_apo antibody_. Paratope allelic polymorphisms with predicted ΔΔG > 10 kcal/mol are shown as 10 kcal/mol. Paratope allelic polymorphisms with predicted ΔΔG < −5 kcal/mol are shown as −5 kcal/mol. See also Figure S1 and Table S1.

To systematically analyze the impact of V-gene allelic polymorphisms on antibody binding activity, we leveraged the collection of antibody-antigen complex structures available in the Structural Antibody Database (SAbDab; http://opig.stats.ox.ac.uk/webapps/sabdab).^20^ Among the 1,048 antibody-antigen complex structures that we analyzed, 544 contained at least one paratope residue with a V-gene allelic polymorphism (see STAR Methods). Most antigens in these 544 structures, which had a median resolution of 2.6 Å (range = 1.2–7.3 Å; Figure S1A), were from viruses, although a considerable number were from human and *Plasmodium* (Figure S1B). Subsequently, we computationally predict the impact of paratope allelic polymorphisms in these 544 structures on antibody binding activity (Figure 1A). For example, there are three allelic polymorphisms at residue 50 of IGHV4-4, namely Arg, Glu, and Tyr. If an antibody is encoded by IGHV4-4 and had V_H_ Arg50 in the paratope, we would predict the effects of V_H_ R50E and V_H_ R50Y on its binding activity (n.b. V_H_ and V_L_ denote heavy- and light-chain residues, respectively). In this study, we predicted the effects of 1,150 paratope allelic polymorphisms across 544 structures on antibody binding activity (see STAR Methods and Table S1).

A paratope allelic polymorphism could impact the stability of the antibody-antigen complex structure (ΔΔG_complex_) by altering the stability of the antibody (ΔΔG_apo antibody_) as well as the antibody-antigen binding energy (ΔΔG_binding_). Therefore, to predict ΔΔG_binding_, we would need to first predict both ΔΔG_complex_ and ΔΔG_apo antibody_ (Figures S1C and S1D). Here, we used FoldX^21^ to predict the ΔΔG_complex_ and ΔΔG_apo antibody_ of each of the 1,150 paratope allelic polymorphisms. The predicted ΔΔG_binding_ was then calculated by subtracting predicted ΔΔG_apo antibody_ from predicted ΔΔG_complex_. Here, ΔΔG > 0 kcal/mol indicated destabilization. Many paratope allelic polymorphisms had a higher predicted ΔΔG_complex_ than the predicted ΔΔG_apo antibody_ (i.e., predicted ΔΔG_binding_ > 0 kcal/mol; Figures 1B and 1C). Of note, predicted ΔΔG_binding_ had minimal correlation with the resolution of the structures (rank correlation = −0.16; Figure S1E), indicating that quality of the structures did not systematically bias the estimation of ΔΔG_binding_.

### Paratope allelic polymorphisms often affect antibody binding activity

A previous benchmarking study has shown that FoldX has a 70% accuracy of classifying whether a mutation is stabilizing (ΔΔG < 0 kcal/mol) or destabilizing (ΔΔG > 0 kcal/mol).^22^ Among the 1,150 paratope allelic polymorphisms from 544 structures, 73% (837 paratope allelic polymorphisms from 464 structures) were predicted to disrupt binding, which consisted of 149 non-redundant V-gene allelic polymorphisms. Of note, 21% and 64% of these paratope allelic polymorphisms that were predicted to disrupt binding were in CDRs H1 and H2, respectively. Although FoldX is known to be more accurate at predicting destabilizing mutations than stabilizing mutations,^23^ we acknowledge that FoldX may misclassify some mutations that improve or have no effect on binding (i.e., ΔΔG_binding_ ≤ 0 kcal/mol) as disruptive (i.e., ΔΔG_binding_ > 0 kcal/mol). At the same time, the impact of certain paratope allelic polymorphisms on antibody binding activity may be underestimated by FoldX. For example, while the binding dissociation constant (K_D_) of IGHV2-5 antibody 2-7 to the receptor-binding domain of SARS-CoV-2 spike was shown to be >100-fold weaker with the IGHV2-5 allelic polymorphism D54N,^6^ its predicted ΔΔG_binding_ was only 0.63 kcal/mol, corresponding to only around a 3-fold change in K_D_ (Figure 1C).

**Figure 2:**
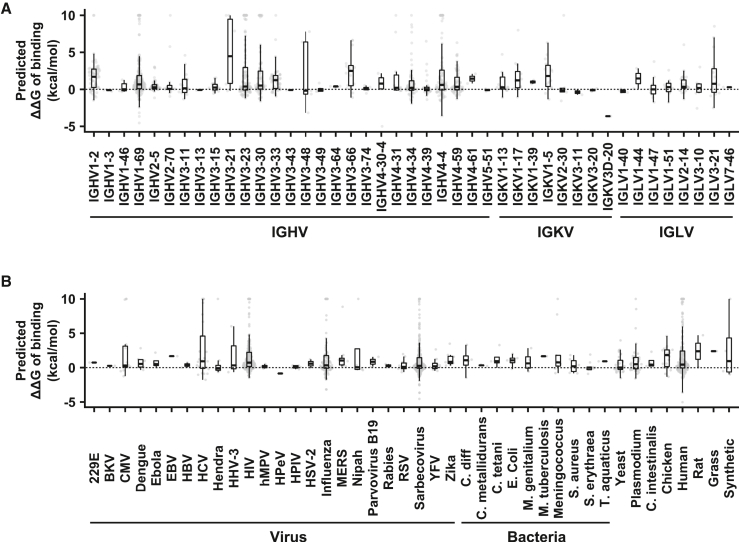
Immunoglobulin V-gene allelic polymorphisms influence the binding activity of diverse antibodies The distributions of predicted ΔΔG_binding_ of paratope allelic polymorphisms in (A) different V genes and (B) antibodies to different antigens are shown. One data point represents one paratope allelic polymorphism. For the boxplot, the middle horizontal line represents the median. The lower and upper hinges represent the first and third quartiles, respectively. The upper whisker extends to the highest data point within a 1.5× inter-quartile range (IQR) of the third quartile, whereas the lower whisker extends to the lowest data point within a 1.5× IQR of the first quartile. Paratope allelic polymorphisms with predicted ΔΔG > 10 kcal/mol are shown as 10 kcal/mol. Paratope allelic polymorphisms with predicted ΔΔG < −5 kcal/mol are shown as −5 kcal/mol. See also Table S1.

Next, we examined whether the impact of allelic polymorphisms on antibody binding activity could be observed in different V genes. Within the 544 antibody-antigen complex structures that were analyzed, paratope allelic polymorphisms were identified in 43 V genes, which represent 33% of the 131 functional V genes in the IMGT database.^24^ Among these 43 V genes, 88% (38/43) had at least one paratope allelic polymorphism with a predicted ΔΔG_binding_ > 0 kcal/mol (Figure 2A). These V genes spanned five heavy-chain V-gene (IGHV) families (IGHV1, IGHV2, IGHV3, IGHV4, IGHV5), three kappa light-chain V-gene (IGKV) families (IGKV1, IGKV2, IGKV3), and four lambda light-chain V-gene (IGLV) families (IGLV1, IGLV2, IGLV3, IGLV7). Similarly, we also observed that paratope allelic polymorphisms affected antibody binding to many different antigens (Figure 2B). For example, 74% (571/776) and 73% (33/45) of paratope allelic polymorphisms in antibodies to viral and bacterial antigens, respectively, were predicted to disrupt binding. We also observed that 72% (168/232) of paratope allelic polymorphisms in antibodies to human proteins were predicted to disrupt binding. These antibodies to human proteins include several FDA-approved therapeutic antibodies, namely avelumab (PDB: 5GRJ),^25^ dupilumab (PDB: 6WGL),^26^ tralokinumab (PDB: 5L6Y),^27^ atezolizumab (PDB: 5XXY),^28^ dostarlimab (PDB: 7WSL),^29^ and daratumumab (PDB: 7DHA).^30^ Overall, these observations suggest that V-gene allelic polymorphisms play a critical role in determining the binding activity of antibodies with different germline usages and specificities.

### Experimentally validating the importance of paratope allelic polymorphisms

**Figure 3:**
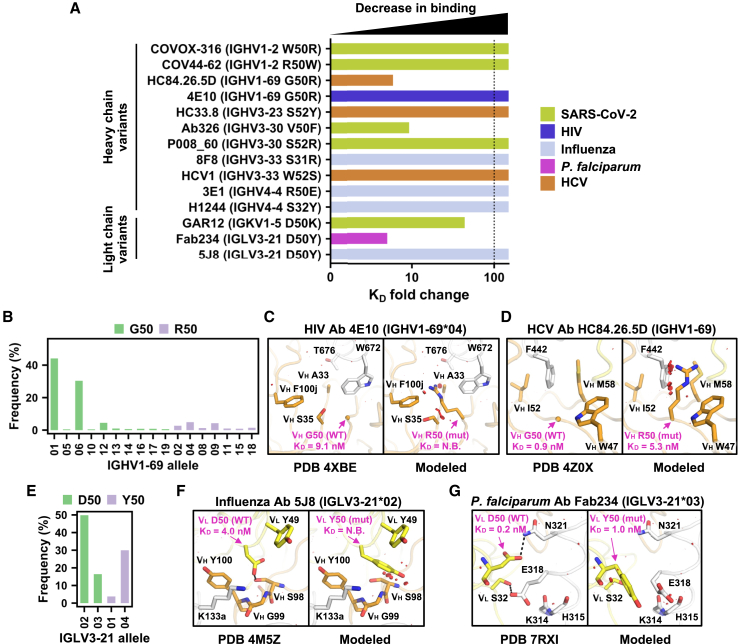
A given allelic polymorphism can impact the binding activity of antibodies to different antigens (A) The impact of the indicated paratope allelic polymorphisms on the antibody binding affinity of the corresponding antibodies is quantified by the fold change in the binding dissociation constant (K_D_). A higher fold change indicates worse binding. Paratope allelic polymorphisms that abolished antibody binding are shown with a fold change of >100. Antibody specificity is color-coded. (B and E) Allele usages of (B) IGHV1-69 antibodies and (E) IGLV3-21 antibodies are shown. The y axis represents the allele frequency among antibodies in GenBank that are encoded by the indicated V gene. Bar color represents the amino acid identity at the indicated residue position. (C, D, F, and G) Structural effects of paratope allelic polymorphisms (C) V_H_ G50R of antibody 4E10 in complex with HIV Env membrane-proximal external region (PDB: 4XBE),^31^ (D) V_H_ G50R of antibody HC84.26.5D in complex with HCV glycoprotein E2 (PDB: 4Z0X),^32^ (F) V_L_ D50Y of antibody 5J8 in complex with influenza H1N1 A/California/07/2009 HA1 subunit (PDB: 4M5Z),^33^ and (G) V_L_ D50Y of Fab234 in complex with C-terminal αTSR domain of *P. falciparum* circumsporozoite protein (PDB: 7RXI)^34^ are modeled by FoldX.^21^ Left: previously determined experimental structures of antibody-antigen complexes. Right: models of alternative allelic polymorphisms. Allelic polymorphic residues are labeled in magenta. K_D_ values of wild-type and allelic mutants to antigens were measured by biolayer interferometry (BLI) and are indicated. N.B. represents no binding. Antibody heavy and light chains are shown in orange and yellow, respectively. Antigens are shown in white. Red disks indicate significant van der Waals overlap (distance < 2.8 Å), hence representing a steric clash. H-bonds are represented by black dashed lines. Kabat numbering is applied to all antibodies. Cα atoms of glycines are represented by spheres. The V-gene and allele usage for each antibody is indicated. Of note, the allele usage for HC84.26.5D cannot be assigned unambiguously. See also Figures S2–S4 and Table S2.

To further confirm the prevalent impact of V-gene allelic polymorphisms in antibody paratopes, we experimentally determined the effect of 14 paratope allelic polymorphisms with a predicted ΔΔG_binding_ > 0 on antibody binding affinity (Table S2). They were selected among antibodies that bind to the antigens of five medically important pathogens, namely SARS-CoV-2 spike, hepatitis C virus (HCV) E2 envelope glycoprotein, HIV envelope glycoprotein, influenza HA, and *P. falciparum* circumsporozoite protein (CSP). In addition, these 14 paratope allelic polymorphisms were on eight different V genes and had different biophysical properties, such as charge reversion (IGHV4-4 R50E), decrease in side-chain volume (IGHV3-33 W52S), and increase in side-chain volume (IGHV3-30 V50F). Our BLI experiment showed that among these 14 paratope allelic polymorphisms, 10 abolished the antibody binding activity, whereas the remaining four weakened the binding affinity by at least 5-fold (Figure 3A; Figures S2 and S4). These findings substantiate that paratope allelic polymorphisms have a prevalent impact on the binding activity of diverse antibodies.

### A given allelic polymorphism can impact diverse antibodies

IGHV1-69 is frequently utilized in the antibody response against microbial pathogens.^35^^,^^36^ Among all of the paratope allelic polymorphisms in IGHV1-69, G50R has an exceptionally high predicted ΔΔG_binding_ across multiple antibodies (Figure S3). IGHV1-69 encodes either Gly or Arg at residue 50, depending on the allele. Among 1,266 IGHV1-69 antibodies from GenBank,^37^ 84% were encoded by alleles with Gly50 (Figure 3B). Previous studies have shown that allelic polymorphism V_H_ G50R would abolish the binding activity of IGHV1-69 antibodies to *S. aureus*.^4^ Our BLI experiment further demonstrated that V_H_ G50R abolished binding of IGHV1-69 antibody 4E10 to the membrane-proximal external region (MPER) of HIV envelope (Env) and reduced binding of another IGHV1-69 antibody, HC84.26.5D, to HCV E2 by around 6-fold (Figure 3A; Figure S2). Of note, 4E10, which was discovered more than 20 years ago,^38^ represents a multidonor class of IGHV1-69/IGKV3-20 broadly neutralizing antibodies to HIV.^39^ Structural modeling showed that V_H_ G50R would introduce substantial steric clash between 4E10 and HIV Env (Figure 3C). A similar observation was made for V_H_ G50R in antibody HC84.26.5D (PDB: 4Z0X)^32^ (Figure 3D). A recent preprint has reported that V_H_ G50R can also abolish the binding activity of another IGHV1-69 antibody to HCV E2,^40^ although its epitope differs from that of HC84.26.5D. These findings demonstrate that multiple IGHV1-69 antibodies in the literature with different specificities have a strong preference toward alleles that encode Gly50 rather than Arg50.

Another example was IGLV3-21 residue 50, which has either Asp or Tyr, depending on the allele. Among 347 IGLV3-21 antibodies from GenBank,^37^ 66% were encoded by alleles with Asp50 (Figure 3E). Our BLI experiment showed that V_L_ D50Y abolished the binding activity of IGLV3-21 antibody 5J8 to influenza H1N1 A/California/07/2009 HA and weakened the binding affinity of another IGLV3-21 antibody, Fab234, to *P. falciparum* CSP by around 5-fold (Figure 3A; Figure S2). Structural modeling indicated that V_L_ D50Y would remove an H-bond and introduce steric clashes between 5J8 and influenza HA (Figure 3F) and, similarly, result in loss of an H-bond between Fab234 and *P. falciparum* CSP (Figure 3G). These observations not only substantiate that a given allelic polymorphism can impact the binding activities of diverse antibodies but also demonstrate that such a phenomenon also arises in light chain.

### Importance of minor allelic polymorphisms in broadly neutralizing antibodies

**Figure 4:**
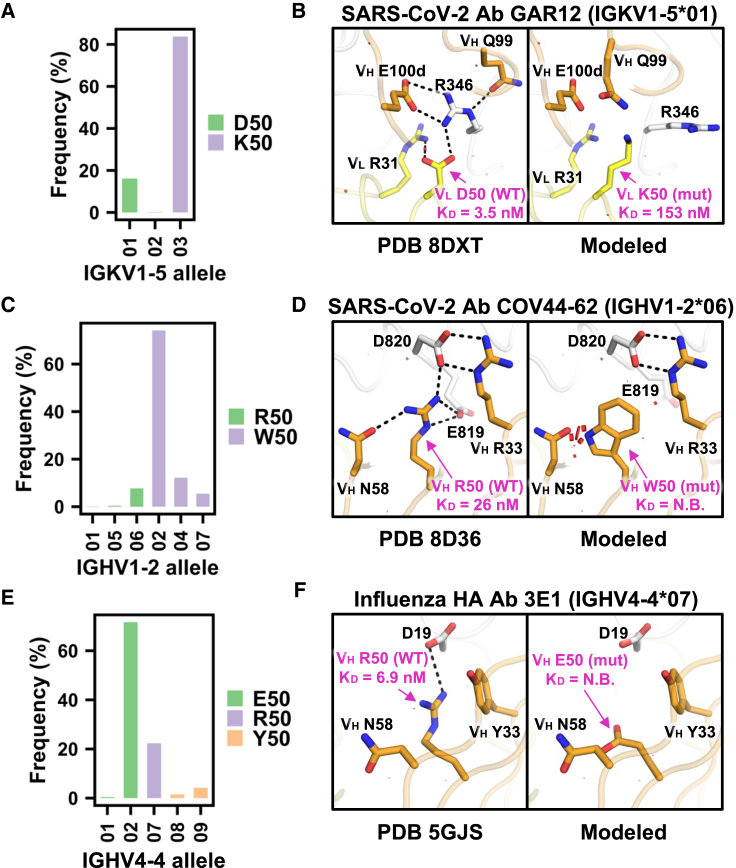
Minor immunoglobulin V-gene allelic polymorphisms are important for the binding activity of diverse antibodies (A, C, and E) Allele usage of (A) IGKV1-5 antibodies, (C) IGHV1-2 antibodies, and (E) IGHV4-4 antibodies is shown. Bar color represents the amino acid identity at the indicated residue position. The y axis represents the allele frequency among antibodies in GenBank that are encoded by the indicated V gene. (B, D, and F) Structural effects of paratope allelic polymorphisms (B) V_L_ D50K of antibody GAR12 in complex with the receptor-binding domain of SARS-CoV-2 spike (PDB: 8DXT),^41^ (D) V_H_ R50W of antibody COV44-62 in complex with the fusion peptide of SARS-CoV-2 spike (PDB: 8D36),^42^ and (F) V_H_ R50E of antibody 3E1 in complex with influenza H1N1 A/California/04/2009 HA (PDB: 5GJS)^43^ are modeled by FoldX.^21^ Structure visualization has the same style and format as Figure 3, with H-bonds and salt bridges represented by black dashed lines. See also Figures S2–S4 and Table S2.

The binding activities of three out of 14 antibodies in our BLI experiment (Figure 3A) were attributed to minor V-gene allelic polymorphisms (i.e., allelic polymorphism frequency <25% among antibodies in GenBank; Table S2). These three antibodies, namely GAR12, COV44-62, and 3E1, are all broadly neutralizing antibodies. GAR12 targets the receptor-binding domain of SARS-CoV-2 spike and was previously shown to neutralize all tested variants of concern, including Omicron BA.1, BA.2, and BA.5.^41^ The light chain of GAR12 is encoded by allele ^∗^01 of IGKV1-5, which has a minor allelic polymorphism Asp50. Among 744 IGKV1-5 antibodies from GenBank,^37^ only 16% were encoded by alleles with Asp50 (allele ^∗^01 or ^∗^02), whereas the remaining 84% were encoded by allele ^∗^03, which had Lys50 (Figure 4A). Our BLI experiment showed that the binding of GAR12 to the receptor-binding domain of SARS-CoV-2 spike was reduced by 44-fold when V_L_ Asp50 was mutated to the major allelic polymorphism V_L_ Lys50. Structural modeling indicated that V_L_ D50K would disrupt an extensive electrostatic interaction network with Arg346 of the receptor-binding domain of SARS-CoV-2 spike and introduce unfavorable electrostatic interactions (Figure 4B).

COV44-62 targets the highly conserved fusion peptide of coronavirus spike and was previously shown to neutralize various coronavirus strains from different genera.^42^ The heavy chain of COV44-62 is encoded by allele ^∗^06 of IGHV1-2, which has a minor allelic polymorphism Arg50. Among 788 IGHV1-2 antibodies from GenBank,^37^ only 8% were encoded by alleles with Arg50, whereas the remaining 92% were encoded by alleles with Trp50 (Figure 4C). Our BLI experiment showed that binding of COV44-62 to the fusion peptide of SARS-CoV-2 spike was abolished by mutating V_H_ Arg50 to the major allelic polymorphism V_H_ Trp50 (Figure 3A; Figure S2). Structural modeling indicated that V_H_ R50W would remove multiple salt bridges between COV44-62 and SARS-CoV-2 fusion peptide as well as introduce steric clashes at the binding interface (Figure 4D).

3E1 targets the conserved stem domain of influenza HA and was previously shown to neutralize influenza A H1 and H5 subtypes.^43^ The heavy chain of 3E1 is encoded by IGHV4-4, which has a minor allelic polymorphism Arg50. Among 264 IGHV4-4 antibodies from GenBank,^37^ 6%, 22%, and 72% were encoded by alleles with Tyr50, Arg50, and Glu50, respectively (Figure 4E). Our BLI experiment showed that the binding of 3E1 to influenza H1N1 A/California/07/2009 HA was abolished by mutating V_H_ Arg50 to the major allelic polymorphism V_H_ Glu50 (Figure 3A; Figure S2). Structural modeling showed that V_H_ R50E would eliminate a salt bridge between 3E1 and influenza HA (Figure 4F). Overall, these results demonstrate the contribution of minor V-gene allelic polymorphisms to broadly neutralizing antibody responses against different antigens.

## Discussion

Previous studies have provided limited examples of how V-gene allelic polymorphisms can affect the binding activity of antibodies of interest.^3^^,^^4^^,^^5^^,^^6^^,^^7^^,^^8^ By analyzing more than a thousand publicly available antibody-antigen complex structures, our study here shows that the impact of V-gene allelic polymorphisms on antibody binding activity is highly prevalent. Among the 1,048 antibody-antigen complex structures being analyzed, 44% (464/1,048) contain allelic polymorphisms that were predicted to disrupt binding. Besides, the binding activity of all tested antibodies (14 out of 14) was decreased or abolished by alternative allelic polymorphisms. Consistent with these findings, a recent preprint reports that V-gene allelic polymorphisms are linked to the variability of antibody repertoires across individuals.^44^ These observations indicate that V-gene allelic polymorphisms can greatly influence antibody responses to different antigens. Consequently, the potency and breadth of antibody response that is elicited by infection or vaccination may be associated with V-gene allele usage. This phenomenon is indeed observed in a phase 1 clinical trial of an HIV vaccine candidate.^8^^,^^9^ Given that certain broadly neutralizing antibodies rely on minor V-gene allelic polymorphisms, V-gene allelic polymorphisms in the human population are an important consideration in the pursuit of more universal vaccines. At the same time, we acknowledge that the binding activity of many antibodies are independent of V-gene allelic polymorphisms. Besides, antibodies with different alleles may prefer different epitopes yet ones on the same antigen.^45^

The need, if any, for maintaining multiple alleles of a given V gene in the human population has been previously regarded as an evolutionary mystery.^2^ One possibility is that different alleles of a given V gene have different antigen-binding preferences, which would in turn lead to a heterozygote advantage. An example is residue 50 of IGHV1-69, which encodes either Gly or Arg, depending on alleles. V_H_ G50R abolished the binding activity of IGHV1-69 antibodies to *S. aureus*, as shown in a previous study,^4^ as well as HIV Env and HCV E2, as shown in our work here. Nevertheless, a recent study has shown that V_H_ Arg50 is essential for IGHV1-69 antibodies to interact with the receptor-binding domain of SARS-CoV-2 spike.^3^ As a result, mounting an optimal IGHV1-69 antibody response against *S. aureus*, HIV, HCV, and SARS-CoV-2 would require both Gly50 and Arg50 alleles of IGHV1-69. In other words, individuals with either Gly50 or Arg50 in all copies of the IGHV1-69 gene may have difficulties generating an effective IGHV1-69 antibody response against certain pathogens. Another example is residue 50 of IGHV1-2, which encodes either Arg or Trp, depending on alleles. Our work here showed that V_H_ Arg50 is essential for the binding activity of COV44-62, which is an IGHV1-2 broadly neutralizing coronavirus antibody.^42^ In contrast, V_H_ Trp50 is essential for the binding activity of IGHV1-2 broadly neutralizing HIV antibodies.^8^^,^^12^^,^^13^ Based on our ΔΔG_binding_ prediction results, similar observations can be made for allelic polymorphisms in other germline genes (Figure S3). Nevertheless, future experimental studies will be needed to fully dissect the evolutionary causes and consequences of allelic polymorphisms in different V genes.

In this study, we also identified several FDA-approved therapeutic antibodies where paratope allelic polymorphisms were predicted to strongly disrupt binding to human proteins, including cancer therapeutic targets. These antibodies were identified by phage display screening of human antibody libraries (e.g., avelumab,^46^ tralokinumab,^47^ and atezolizumab^48^) and immunization of humanized mice (e.g., dupilumab^49^ and daratumumab^50^), which are two common methods for antibody discovery. Both methods require cloning of antibody repertoires from human donors. Based on our results, it is likely that the probability of success of antibody discovery through phage display screening and humanized mice depends not only on the antigens but also on the V-gene allelic polymorphisms of the human donors present in the phage-displayed antibody libraries or in the humanized mice. Future antibody discovery may benefit from having multiple human donors with diverse V gene alleles. Given that the market size of monoclonal antibodies continues to grow,^51^ understanding the impact of V-gene allelic polymorphisms on antibody discovery will have important public health implications.

While this study indicates that many V-gene allelic polymorphisms can influence antibody binding activity, our analysis only focused on those in the paratope. Previous studies have demonstrated that non-paratope mutations can also affect antibody binding activity.^52^^,^^53^^,^^54^ Therefore, some non-paratope V-gene allele polymorphisms may possibly do the same. Besides, the diversity of human V-gene alleles is likely higher than what is currently known. Over the past decade, numerous V-gene alleles have been discovered thanks to the advances in computational methods for germline gene inference and long-read third-generation sequencing technologies.^19^ The ongoing efforts in understanding the diversity of V genes across human populations and ethnicities, including geographic diversity, will likely reveal many more novel V-gene alleles.^55^ Of note, the impact of V-gene allelic polymorphisms on antibody binding are unlikely to be limited to human V genes since a similar observation has been recently reported in IGHV3-73 of rhesus macaque to SARS-CoV-2.^56^ Together, it is likely that V-gene allelic polymorphisms, as well as their impact on antibody binding activity, are more widespread than indicated by our and previous studies.^19^

### Limitations of the study

For antibodies to pathogens, the existing antibody-antigen complex structures are heavily biased toward those with neutralization activity. It is unclear whether V-gene allelic polymorphisms also have a prevalence effect on the binding activity of non-neutralizing antibodies. In addition, although FoldX has a decent accuracy in identifying destabilizing mutations,^22^^,^^23^ it only has moderate performance in predicting the magnitude of effect on binding activity.^22^ Therefore, caution is needed to infer the change in K_D_ from the predicted ΔΔG_binding_. Moreover, our study mostly focused on affinity-matured antibodies, including all those in our experimental validation (Figure 3A; Table S2). While we anticipate that the effects of allelic polymorphisms on binding activity should also apply to their corresponding unmutated common ancestor (UCA), experimental confirmation is warranted in future studies. Lastly, this study estimated the frequency of different V-gene alleles using the antibody sequences in GenBank, which came from more than 200 different studies. Although such estimation may deviate from that in the human population due to the bias in sequence deposition, the frequency of different V-gene alleles in various ethnic and geographical backgrounds remains to be comprehended.^55^

## STAR★Methods

### Key resources table

**Table:** 

REAGENT or RESOURCE	SOURCE	IDENTIFIER
**Chemicals, peptides, and recombinant proteins**		

NEBuilder HiFi DNA Assembly Master Mix	New England Biolabs	Cat#E2621L
FuGENE HD Transfection Reagent	FuGENE	#E231A
ExpiCHO Expression System Kit	Gibco	#A29133
Expi293 Expression System Kit	Gibco	#A14635
SARS-CoV-2 spike and RBD proteins	In house	N/A
H1N1 A/California/07/2009 HA protein	In house	N/A
H1N1 A/Beijing/262/1995 HA protein	In house	N/A
H2N2 A/Japan/305/1957 HA protein	In house	N/A
HCV (isolate H77) E2 domain protein	In house	N/A
HIV Env MPER peptide	GenScript	N/A
SARS-CoV-2 fusion peptide	GenScript	N/A
Opti-MEM Reduced Serum Medium	Thermo Fisher Scientific	Cat#31985070
FreeStyle 293 expression medium	GIBCO	Cat#12338002
Insect-XPRESS Protein-free Insect Cell Medium with L-glutamine	Lonza	Cat#BP12-730Q
PEI MAX transfection reagent	Polysciences	Cat#24765-1
SARS-CoV-2 fusion peptide	GenScript	N/A

**Critical commercial assays**		

Octet NTA Biosensors	Sartorius	Cat#18-5101
Octet Streptavidin (SA) Biosensor	Sartorius	Cat#18-5019
Octet Anti-Human Fab-CH1 2ND Generation (FAB2G) Biosensors	Sartorius	Cat#18-5125

**Experimental models: Cell lines**		

ExpiCHO cells	Thermo Fisher Scientific	Cat#A29127; RRID: CVCL_5J31
Expi293F cells	Thermo Fisher Scientific	Cat#A14527; RRID: CVCL_D615
FreeStyle HEK293F cells	Gibco	Cat#R79007
Sf9 cells	ATCC	RRID: CVCL_0549
High Five Cells	Thermo Fisher Scientific	RRID: CVCL_C190

**Recombinant DNA**		

phCMV3	Genlantis	Cat#P003300
pFastBac	Ekiert et al.^57^	N/A

**Software and algorithms**		

Python	https://www.python.org/	N/A
R	https://www.r-project.org/	N/A
V-Quest online tool	IMGT	https://imgt.org/
FoldX	FoldX Suite	https://foldxsuite.crg.eu/
PyMOL	PyMOL by Schrödinger	https://pymol.org
Custom scripts	This study	https://doi.org/10.5281/zenodo.8330193

**Other**		

0.2 μm membrane filters	Fisher Scientific	Cat#564-0020
HisPur Ni-NTA Resin	Thermo Fisher Scientific	Cat#88221
CaptureSelect CH1-XL Pre-packed Column	Thermo Fisher Scientific	Cat#494346201
Superdex 200 Increase10/300 GL column	GE Healthcare	Cat#GE28-9909-44
Amicon tubes (100K, 30K, 10K)	Millipore	Cat#UFC9100, Cat#UFC9030, Cat#UFC9010
Bio-One Polypropylene 96-well F-Bottom Microplates	Greiner	Cat#655209

### Resource availability

#### Lead contact

Information and requests for resources should be directed to and will be fulfilled by the lead contact, Nicholas C. Wu (nicwu@illinois.edu).

#### Materials availability

All plasmids generated in this study are available from the lead contact without restriction.

### Experimental models and subject details

#### Cell cultures

FreeStyle HEK293F cells (human embryonic kidney cells, female), ExpiCHO cells (Chinese hamster ovary cells, female) and Expi293F cells (human embryonic kidney cells, female) were maintained in FreeStyle 293 expression medium, ExpiCHO expression medium, and Expi293 expression medium, respectively, at 37°C with 8% CO_2_ according to the manufacturer’s instructions (Thermo Fisher Scientific). Sf9 cells (*Spodoptera frugiperda* ovarian cells, female) and High Five cells (*Trichoplusia ni* ovarian cells, female) were maintained in Insect-XPRESS medium (Lonza).

### Method details

#### Identification of paratope residues with allelic polymorphisms

A total of 3,240 human antibody-antigen complex structures were downloaded as PDB files from the Structural Antibody Database (SAbDab, http://opig.stats.ox.ac.uk/webapps/sabdab).^20^ Next, PDB files with more than one antibody were filtered out, leaving 1,416 complex structures. We further discarded the following PDB files due to formatting issues: 7T1W, 7T1X, 6TUL, 6SS4, 6SS5, 7DWT, 7DWU, 6SS2, 6ZJG, 7T0W, 6YXM, 6TKF, 6TKE, 6TKD, 6TKC, 3J6U, 7R8U, and 6YXL, leaving 1,048 complex structures. For each PDB file, an apo antibody structure was generated by removing the antigen from the PDB file. Relative solvent accessibility (RSA) for each antibody residue, either in apo form or in complex with antigen, was computed by DSSP.^58^ Residues with a higher RSA value in the apo antibody structure than the complex structure (i.e., RSA_apo antibody_ – RSA_complex_ > 0) were defined as paratope residues. Germline sequences for human IGHV and IGK(L)V genes were downloaded from the IMGT database (https://www.imgt.org/)^24^ on November 25, 2021. Each position of the antibody sequences in the PDB files and the germline immunoglobulin V gene sequences was numbered according to Kabat numbering using ANARCI.^59^

The germline V genes of each antibody were identified using PyIR,^60^ a wrapper for the IgBLAST.^61^ For each paratope residue, its allelic polymorphisms, if any, across different alleles of the corresponding germline V gene were compared. Paratope residues with no allelic polymorphism were excluded from downstream analysis. In addition, if the amino acid sequence of a given paratope residue was not germline-encoded, such a paratope residue was also discarded. If a paratope residue has two alternative allelic polymorphisms, both were investigated. In summary, a total of 1,150 paratope allelic polymorphisms across 544 antibody-antigen complex structures were identified. Of note, alleles were not reported for antibodies with ambiguity in allele assignment (e.g., HC84.26.5D in Figure 3D).

#### Predicting the ΔΔG of binding for allelic polymorphisms

The ΔΔG for each paratope allelic polymorphisms was predicted using FoldX.^21^ For each paratope allelic polymorphism of a given antibody, two ΔΔG values were predicted, one for the apo antibody structure (ΔΔG_apo antibody_) and the other for the antibody-antigen complex structure (ΔΔG_complex_). Apo antibody structures were generated by extracting the antibody coordinates from the PDB files. Predicted ΔΔG of antibody-antigen binding (ΔΔG_binding_) was computed as:

$$\text{Predicted}\;{\Delta \Delta G}_{binding}=\text{Predicted}\;{\Delta \Delta G}_{complex}-{\text{Predicted}\;\Delta \Delta G}_{apo\;antibody}$$

#### Germline immunoglobulin V gene and allele assignment of antibodies from GenBank

The germline immunoglobulin V genes and alleles of 12,487 antibodies from GenBank (www.ncbi.nlm.nih.gov/genbank)^37^ were identified using PyIR.^60^ Antibodies with ambiguity in allele assignment were excluded from our analysis.

#### Expression and purification of fabs

The heavy and light chains of Fabs were cloned into phCMV3 vector. PCR-based mutagenesis was performed to generate the alternative allelic polymorphisms. The plasmids were transiently co-transfected into ExpiCHO cells at a ratio of 2:1 (heavy chain to light chain) using ExpiFectamine CHO Reagent (Thermo Fisher Scientific) according to the manufacturer’s instructions. The supernatant was collected at 7 days post-transfection. The Fab was purified with a CaptureSelect CH1-XL Pre-packed Column (Thermo Fisher Scientific), followed by a buffer exchange to Dulbecco’s Phosphate Buffered Saline (PBS, pH 7.4).

#### Expression and purification of antigens

SARS-CoV-2 receptor-binding domain was expressed in High Five cells and purified with Ni-NTA resin followed by size exclusion as described previously.^62^ SARS-CoV-2 whole spike protein HexaPro was a gift from Jason McLellan (Addgene plasmid # 154754). SARS-CoV-2 HexaPro was expressed in Expi293F cells and purified with Ni-NTA resin followed by size exclusion as described previously.^63^ The influenza hemagglutinin (HA) proteins from H1N1 A/California/07/2009, H1N1 A/Beijing/262/1995, and H2N2 A/Japan/305/1957 were expressed in High Five cells, purified with Ni-NTA resin followed by size exclusion, and biotinylated as described previously.^57^ The HCV (isolate H77) E2 domain was expressed in HEK293S cells and purified as described previously.^64^ The biotinylated SARS-CoV-2 fusion peptide (N′-biotin-DPSKPSKRSFIEDLLFNKVT-C′) and His-tagged HIV Env MPER peptide (N′-NWFDITNWLWYIKSGGSHHHHHHHH-C′) were chemically synthesized by GenScript.

#### Biolayer interferometry (BLI) binding assays

Binding assays were performed by biolayer interferometry (BLI) using an Octet Red instrument (FortéBio). 20 μg/mL of antibodies, antigens, or peptides in 1x kinetics buffer (1x PBS, pH 7.4, 0.01% BSA and 0.002% Tween 20) were loaded onto different types of sensors, and then incubated with 33 nM, 100 nM, and 300 nM of binders. All antibodies were in Fab format. Specifically, biotinylated SARS-CoV-2 fusion peptide was loaded onto Streptavidin (SA) sensors and incubated with COV44-62 (WT or V_H_ R50W). Biotinylated αTSR domain of *P. falciparum* was loaded onto SA sensors and incubated with Fab234 (WT or V_L_ D50Y). Biotinylated influenza HA from H1N1 A/California/07/2009 was loaded onto SA sensors and incubated with 3E1 (WT or V_H_ R50E), or 5J8 (WT or V_L_ D50). Biotinylated influenza HA from H1N1 A/Beijing/262/1995 was loaded onto SA sensors and incubated with H1244 (WT or V_H_ S32Y). Biotinylated virus HA from H2N2 A/Japan/305/1957 was loaded onto SA sensors and incubated with 8F8 (WT or V_H_ S31R). His_6_-tagged SARS-CoV-2 receptor-binding domain was loaded onto Ni-NTA sensors and incubated with GAR12 (WT or V_L_ D50K), Ab326 (WT or V_H_ V50F), or COVOX-316 (WT or V_H_ W50R). His-tagged HIV Env MPER peptide was loaded onto Ni-NTA sensors and incubated with 4E10 (WT or V_H_ G50R). HC33.8 (WT or V_H_ S52Y), HC84.26.5D (WT or V_H_ G50R), or HCV1 (WT or V_H_ W52S) was loaded onto Anti-Human Fab-CH1 2nd Generation (FAB2G) sensors and incubated with HCV E2. P008_60 (WT or V_H_ S52R) was loaded onto FAB2G sensors and incubated with SARS-CoV-2 spike protein.^63^ The assay consisted of five steps: 1) baseline; 2) loading; 3) baseline; 4) association; and 5) dissociation. For estimating the K_D_ values, a 1:1 binding model was used.

### Quantification and statistical analysis

Standard deviation for K_D_ estimation was computed by Octet analysis software 9.0. Spearman’s rank correlation coefficient was computed in R.

## Data Availability

• Information on the 1,150 paratope allelic polymorphisms that were analyzed in this study are in Table S1. • Custom python scripts for analyzing the deep mutational scanning data have been deposited to https://doi.org/10.5281/zenodo.8330193. • Any additional information required to reanalyze the data reported in this paper is available from the lead contact upon request.

## Supplemental information

Document S1. Figures S1‒S4 and Table S2

Table S1. Information on the 1,150 paratope allelic polymorphisms that were analyzed in this study, related to Figures 1 and 2

Document S2. Article plus supplemental information
