# Supplementary material for: Widespread impact of immunoglobulin V-gene allelic polymorphisms on antibody reactivity
Source: Cell Rep. 2023 Sep 30;42(10):113194. doi: 10.1016/j.celrep.2023.113194 (PMC10636607; doi:10.1016/j.celrep.2023.113194)
Supplement: Document S2. Article plus supplemental information [file mmc3.pdf]

Cell Reports

Article

Widespread impact of immunoglobulin V-gene allelic polymorphisms on antibody reactivity

Graphical abstract

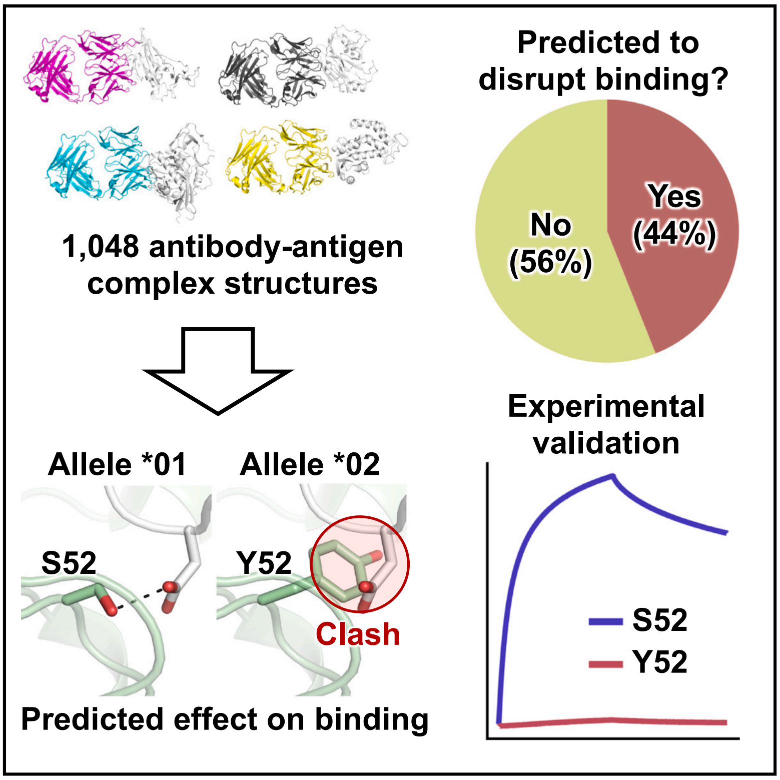

Highlights

- Analysis of V-gene allelic polymorphisms in 1,048 antibody-antigen complex structures
- Many antibodies contain allelic polymorphisms predicted to disrupt binding
- These include antibodies with different V genes and to different pathogens
- Some broadly neutralizing antibodies depend on minor V-gene allelic polymorphisms

Authors

Meng Yuan, Ziqi Feng, Huibin Lv, ..., Logan Talmage, Ian A. Wilson, Nicholas C. Wu

Correspondence

nicwu@illinois.edu

In brief

By analyzing >1,000 publicly available antibody-antigen complex structures, Yuan et al. demonstrate that antibody binding activity is often influenced by V-gene allelic polymorphisms. This result provides mechanistic insights into the variability of antibody repertoires across individuals, which in turn have important implications for vaccine development and antibody discovery.

Yuan et al., 2023, Cell Reports *42*, 113194
October 31, 2023 © 2023 The Author(s).
https://doi.org/10.1016/j.celrep.2023.113194

CellPress

Cell Reports

CellPress
OPEN ACCESS

Article

Widespread impact of immunoglobulin V-gene allelic polymorphisms on antibody reactivity

Meng Yuan,[1] Ziqi Feng,[1] Huibin Lv,[2,3] Natalie So,[2,4] Ivana R. Shen,[2] Timothy J.C. Tan,[5] Qi Wen Teo,[2,3] Wenhao O. Ouyang,[2] Logan Talmage,[2] Ian A. Wilson,[1,6] and Nicholas C. Wu[2,3,5,7,8,*]

[1]Department of Integrative Structural and Computational Biology, The Scripps Research Institute, La Jolla, CA 92037, USA
[2]Department of Biochemistry, University of Illinois at Urbana-Champaign, Urbana, IL 61801, USA
[3]Carl R. Woese Institute for Genomic Biology, University of Illinois at Urbana-Champaign, Urbana, IL 61801, USA
[4]Department of Computer Science, University of Illinois at Urbana-Champaign, Urbana, IL 61801, USA
[5]Center for Biophysics and Quantitative Biology, University of Illinois at Urbana-Champaign, Urbana, IL 61801, USA
[6]The Skaggs Institute for Chemical Biology, The Scripps Research Institute, La Jolla, CA 92037, USA
[7]Carle Illinois College of Medicine, University of Illinois at Urbana-Champaign, Urbana, IL 61801, USA
[8]Lead contact
*Correspondence: nicwu@illinois.edu
https://doi.org/10.1016/j.celrep.2023.113194

SUMMARY

The ability of the human immune system to generate antibodies to any given antigen can be strongly influenced by immunoglobulin V-gene allelic polymorphisms. However, previous studies have provided only limited examples. Therefore, the prevalence of this phenomenon has been unclear. By analyzing >1,000 publicly available antibody-antigen structures, we show that many V-gene allelic polymorphisms in antibody paratopes are determinants for antibody binding activity. Biolayer interferometry experiments further demonstrate that paratope allelic polymorphisms on both heavy and light chains often abolish antibody binding. We also illustrate the importance of minor V-gene allelic polymorphisms with low frequency in several broadly neutralizing antibodies to severe acute respiratory syndrome coronavirus 2 (SARS-CoV-2) and influenza virus. Overall, this study not only highlights the pervasive impact of V-gene allelic polymorphisms on antibody binding but also provides mechanistic insights into the variability of antibody repertoires across individuals, which in turn have important implications for vaccine development and antibody discovery.

INTRODUCTION

Human antibodies, which are produced by B cells and composed of two chains (heavy and light), are central to the immune response against pathogen infection. To be able to recognize many different pathogens, the human antibody repertoire has enormous diversity, with up to 10^{15} unique antibody clonotypes.[1] This diversity is generated mainly by V(D)J recombination, which is a somatic recombination process that assembles different germline gene segments, known as variable (V), diversity (D), and joining (J) genes, into the variable region of the antibody molecule. In addition, many V-gene segments are known to have multiple alleles that encode amino acid differences, which further increase the diversity of human antibody repertoire at the population level.

Allelic polymorphisms of immunoglobulin V genes (referred to as “V genes” hereafter) are enriched in the complementarity-determining regions (CDRs),[2] which usually form the antibody paratopes (i.e., regions that involve in binding to antigens). Of note, allelic polymorphisms in this study refer to polymorphisms at the amino acid sequence level for convenience. Several studies have reported the importance of V-gene allelic polymorphisms in antibody binding. For example, allelic polymorphisms at IGHV1-69 residues 50 (G/R) and 54 (F/L) (n.b. Kabat numbering used throughout for all antibody residues) can influence antibody binding to severe acute respiratory syndrome coronavirus 2 (SARS-CoV-2).[3] Allelic polymorphisms at IGHV1-69 residues 50 (G/R) can also impact antibody binding to *Staphylococcus aureus* (*S. aureus*).[4] Other examples include IGHV3-33 residue 52 (W/S) in antibodies to *Plasmodium falciparum* (*P. falciparum*)[5] and IGHV2-5 residue 54 (D/N) in antibodies to SARS-CoV-2[6] and human immunodeficiency virus (HIV).[7] Therefore, it is apparent that V-gene allelic polymorphisms can affect antibody binding. However, it remains unclear whether the impact of V-gene allelic polymorphisms on antibody binding is prevalent because previous studies typically characterized a single antibody-antigen pair at a time and the number of such studies is rather limited. Furthermore, previous studies of V-gene allelic polymorphisms mainly focused on the heavy chain,[3–10] whereas those within the light chain remain largely unexplored.

Nevertheless, V-gene allelic polymorphisms are shown to have important public health relevance. For instance, IGHV1-2 allelic usage correlates with the response rate to an HIV vaccine candidate in a phase 1 clinical trial (ClinicalTrials.gov: NCT03547245),[8,9,11] due to the influence of its allelic

This is an open access article under the CC BY license (http://creativecommons.org/licenses/by/4.0/).

polymorphisms at residue 50 (W/R) on antibody binding to HIV.[8,12,13] A previous study has also shown that IGHV1-69 allelic usage correlates with the broadly neutralizing antibody response to influenza virus in a vaccine cohort.[14] This observation has also been attributed to the differential autoreactive propensities of different IGHV1-69 alleles,[10] as well as a potentially minor effect of its allelic polymorphisms at residue 54 (L/F) on antibody binding to the conserved stem domain of influenza hemagglutinin (HA).[15,16] Similarly, allele usages of IGHV3-66 and IGHV4-61 were associated with Kawasaki disease[17] and rheumatic heart disease,[18] respectively, although the underlying mechanisms are unknown. As a result, investigating the impact of V-gene allelic polymorphisms can provide critical insights into vaccine development and autoimmune diseases.[19]

In this study, we systematically investigate the effect of V-gene allelic polymorphisms on antibody binding by analyzing 1,048 publicly available antibody-antigen complex structures. V-gene allelic polymorphisms could be identified in the antibody paratope of 52% (544/1,048) complex structures. Computational analysis of protein mutational stability predicted that 73% of paratope allelic polymorphisms (i.e., mutating a paratope residue to alternative allelic polymorphisms) would disrupt antibody binding activity. The wide impact of V-gene allelic polymorphisms on antibody binding was further validated using biolayer interferometry (BLI). Our results also illustrated the importance of light-chain V-gene allelic polymorphisms. In addition, we identified several V-gene allelic polymorphisms that are essential for the binding activity of broadly neutralizing antibodies to SARS-CoV-2 and influenza virus yet have low frequency among antibodies in GenBank. These discoveries suggested that the importance of allelic polymorphisms on antibody reactivity may have been previously underestimated.

RESULTS

Predicting V-gene allelic polymorphisms that weaken antibody binding activity

To systematically analyze the impact of V-gene allelic polymorphisms on antibody binding activity, we leveraged the collection of antibody-antigen complex structures available in the Structural Antibody Database (SAbDab; http://opig.stats.ox.ac.uk/webapps/sabdab).[20] Among the 1,048 antibody-antigen complex structures that we analyzed, 544 contained at least one paratope residue with a V-gene allelic polymorphism (see STAR Methods). Most antigens in these 544 structures, which had a median resolution of 2.6 Å (range = 1.2–7.3 Å; Figure S1A), were from viruses, although a considerable number were from human and *Plasmodium* (Figure S1B). Subsequently, we computationally predict the impact of paratope allelic polymorphisms in these 544 structures on antibody binding activity (Figure 1A). For example, there are three allelic polymorphisms at residue 50 of IGHV4-4, namely Arg, Glu, and Tyr. If an antibody is encoded by IGHV4-4 and had V_H Arg50 in the paratope, we would predict the effects of V_H R50E and V_H R50Y on its binding activity (n.b. V_H and V_L denote heavy- and light-chain residues, respectively). In this study, we predicted the effects of 1,150 paratope allelic polymorphisms across 544 structures on antibody binding activity (see STAR Methods and Table S1).

A paratope allelic polymorphism could impact the stability of the antibody-antigen complex structure ($\Delta\Delta G_{complex}$) by altering the stability of the antibody ($\Delta\Delta G_{apo\ antibody}$) as well as the antibody-antigen binding energy ($\Delta\Delta G_{binding}$). Therefore, to predict $\Delta\Delta G_{binding}$, we would need to first predict both $\Delta\Delta G_{complex}$ and $\Delta\Delta G_{apo\ antibody}$ (Figures S1C and S1D). Here, we used FoldX[21] to predict the $\Delta\Delta G_{complex}$ and $\Delta\Delta G_{apo\ antibody}$ of each of the 1,150 paratope allelic polymorphisms. The predicted $\Delta\Delta G_{binding}$ was then calculated by subtracting predicted $\Delta\Delta G_{apo\ antibody}$ from predicted $\Delta\Delta G_{complex}$. Here, $\Delta\Delta G > 0$ kcal/mol indicated destabilization. Many paratope allelic polymorphisms had a higher predicted $\Delta\Delta G_{complex}$ than the predicted $\Delta\Delta G_{apo\ antibody}$ (i.e., predicted $\Delta\Delta G_{binding} > 0$ kcal/mol; Figures 1B and 1C). Of note, predicted $\Delta\Delta G_{binding}$ had minimal correlation with the resolution of the structures (rank correlation = −0.16; Figure S1E), indicating that quality of the structures did not systematically bias the estimation of $\Delta\Delta G_{binding}$.

Paratope allelic polymorphisms often affect antibody binding activity

A previous benchmarking study has shown that FoldX has a 70% accuracy of classifying whether a mutation is stabilizing ($\Delta G < 0$ kcal/mol) or destabilizing ($\Delta G > 0$ kcal/mol).[22] Among the 1,150 paratope allelic polymorphisms from 544 structures, 73% (837 paratope allelic polymorphisms from 464 structures) were predicted to disrupt binding, which consisted of 149 nonredundant V-gene allelic polymorphisms. Of note, 21% and 64% of these paratope allelic polymorphisms that were predicted to disrupt binding were in CDRs H1 and H2, respectively. Although FoldX is known to be more accurate at predicting destabilizing mutations than stabilizing mutations,[23] we acknowledge that FoldX may misclassify some mutations that improve or have no effect on binding (i.e., $\Delta\Delta G_{binding} \leq 0$ kcal/mol) as disruptive (i.e., $\Delta\Delta G_{binding} > 0$ kcal/mol). At the same time, the impact of certain paratope allelic polymorphisms on antibody binding activity may be underestimated by FoldX. For example, while the binding dissociation constant (K_D) of IGHV2-5 antibody 2-7 to the receptor-binding domain of SARS-CoV-2 spike was shown to be >100-fold weaker with the IGHV2-5 allelic polymorphism D54N,[6] its predicted $\Delta\Delta G_{binding}$ was only 0.63 kcal/mol, corresponding to only around a 3-fold change in K_D (Figure 1C).

Next, we examined whether the impact of allelic polymorphisms on antibody binding activity could be observed in different V genes. Within the 544 antibody-antigen complex structures that were analyzed, paratope allelic polymorphisms were identified in 43 V genes, which represent 33% of the 131 functional V genes in the IMGT database.[24] Among these 43 V genes, 88% (38/43) had at least one paratope allelic polymorphism with a predicted $\Delta\Delta G_{binding} > 0$ kcal/mol (Figure 2A). These V genes spanned five heavy-chain V-gene (IGHV) families (IGHV1, IGHV2, IGHV3, IGHV4, IGHV5), three kappa light-chain V-gene (IGKV) families (IGKV1, IGKV2, IGKV3), and four lambda light-chain V-gene (IGLV) families (IGLV1, IGLV2, IGLV3, IGLV7). Similarly, we also observed that paratope allelic polymorphisms affected antibody binding to many different antigens (Figure 2B). For example, 74% (571/776) and 73% (33/45) of paratope allelic polymorphisms in antibodies to viral and bacterial antigens, respectively, were predicted to disrupt binding. We also

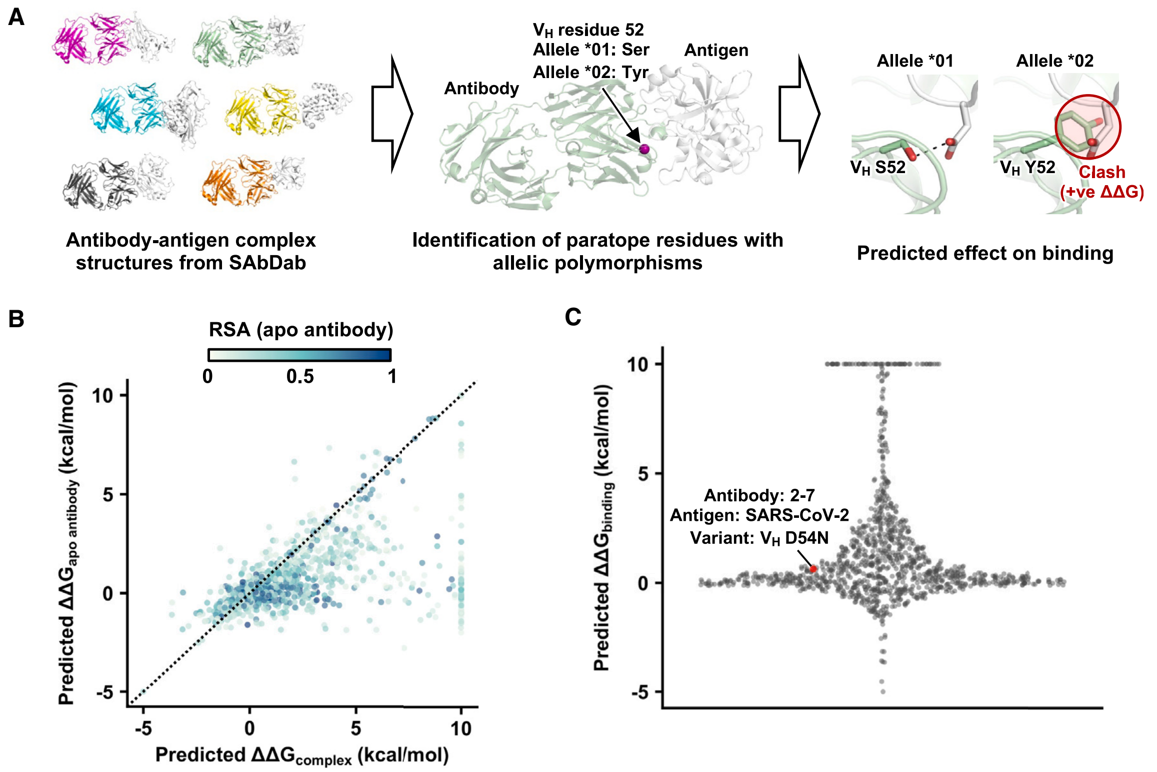

Figure 1. Predicting the effects of immunoglobulin V-gene allelic polymorphisms on antibody binding activity

(A) Schematic of the analysis workflow (see STAR Methods). Briefly, human antibody-antigen complex structures were downloaded as PDB files from the Structural Antibody Database (SAbDab; http://opig.stats.ox.ac.uk/webapps/sabdab).[20] Among these antibodies, paratope residues with allelic polymorphisms were identified. The effects of paratope allelic polymorphisms on antibody binding activity were then predicted using FoldX.[21]

(B) The relationship between predicted $\Delta\Delta G_{\text{apo antibody}}$ and predicted $\Delta\Delta G_{\text{complex}}$ is shown. Each data point is colored by the relative solvent accessibility (RSA) in the apo antibody. Residues that are fully solvent exposed have an RSA of 1, whereas those that are fully buried have an RSA of 0.

(C) The distribution of predicted $\Delta\Delta G_{\text{binding}}$ of 1,150 paratope allelic polymorphisms of 544 antibody-antigen complex structures is shown. Predicted $\Delta\Delta G_{\text{binding}}$ was computed by predicted $\Delta\Delta G_{\text{complex}}$ − predicted $\Delta\Delta G_{\text{apo antibody}}$. Paratope allelic polymorphisms with predicted $\Delta\Delta G > 10$ kcal/mol are shown as 10 kcal/mol. Paratope allelic polymorphisms with predicted $\Delta\Delta G < -5$ kcal/mol are shown as −5 kcal/mol.

See also Figure S1 and Table S1.

observed that 72% (168/232) of paratope allelic polymorphisms in antibodies to human proteins were predicted to disrupt binding. These antibodies to human proteins include several FDA-approved therapeutic antibodies, namely avelumab (PDB: 5GRJ),[25] dupilumab (PDB: 6WGL),[26] tralokinumab (PDB: 5L6Y),[27] atezolizumab (PDB: 5XXY),[28] dostarlimab (PDB: 7WSL),[29] and daratumumab (PDB: 7DHA).[30] Overall, these observations suggest that V-gene allelic polymorphisms play a critical role in determining the binding activity of antibodies with different germline usages and specificities.

Experimentally validating the importance of paratope allelic polymorphisms

To further confirm the prevalent impact of V-gene allelic polymorphisms in antibody paratopes, we experimentally determined the effect of 14 paratope allelic polymorphisms with a predicted $\Delta\Delta G_{\text{binding}} > 0$ on antibody binding affinity (Table S2). They were selected among antibodies that bind to the antigens of five medically important pathogens, namely SARS-CoV-2 spike, hepatitis C virus (HCV) E2 envelope glycoprotein, HIV envelope glycoprotein, influenza HA, and *P. falciparum* circumsporozoite protein (CSP). In addition, these 14 paratope allelic polymorphisms were on eight different V genes and had different biophysical properties, such as charge reversion (IGHV4-4 R50E), decrease in side-chain volume (IGHV3-33 W52S), and increase in side-chain volume (IGHV3-30 V50F). Our BLI experiment showed that among these 14 paratope allelic polymorphisms, 10 abolished the antibody binding activity, whereas the remaining four weakened the binding affinity by at least 5-fold (Figure 3A; Figures S2 and S4). These findings substantiate that paratope allelic polymorphisms have a prevalent impact on the binding activity of diverse antibodies.

A given allelic polymorphism can impact diverse antibodies

IGHV1-69 is frequently utilized in the antibody response against microbial pathogens.[35,36] Among all of the paratope allelic polymorphisms in IGHV1-69, G50R has an exceptionally high predicted $\Delta\Delta G_{\text{binding}}$ across multiple antibodies (Figure S3). IGHV1-69 encodes either Gly or Arg at residue 50,

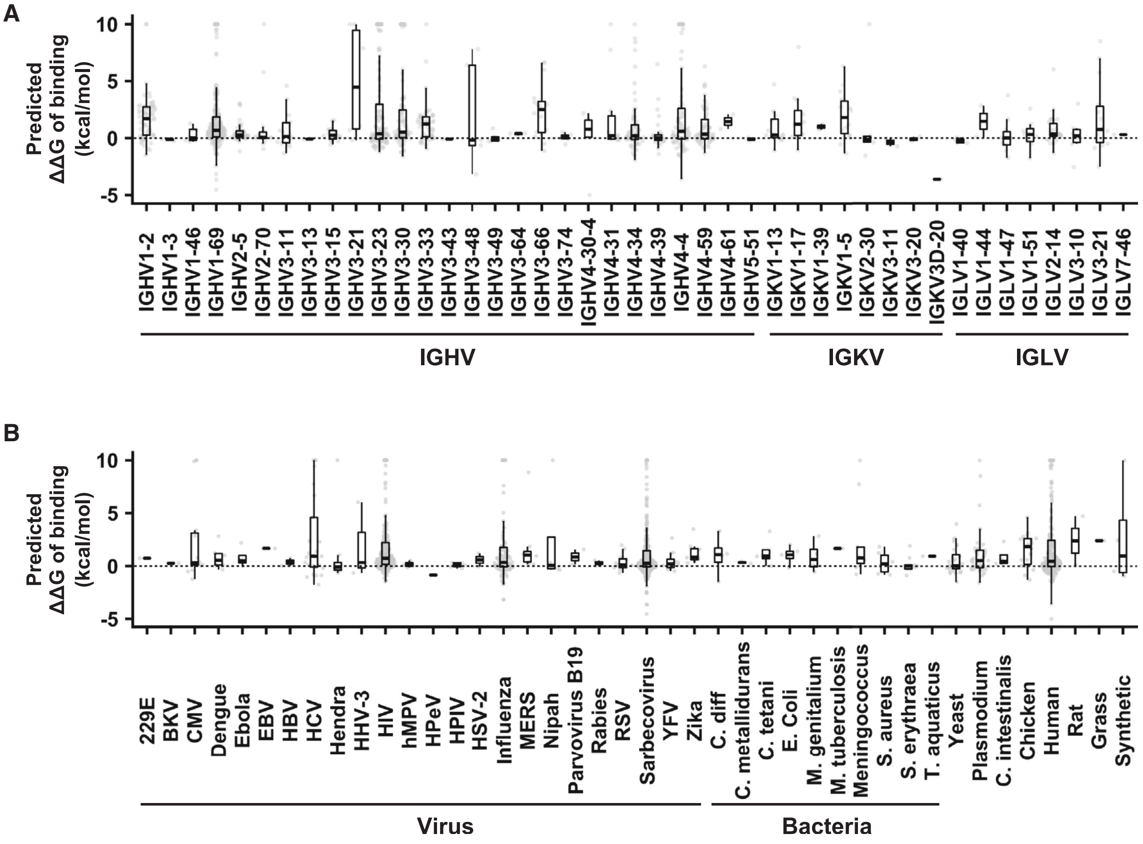

Figure 2. Immunoglobulin V-gene allelic polymorphisms influence the binding activity of diverse antibodies

The distributions of predicted $\Delta\Delta G_{binding}$ of paratope allelic polymorphisms in (A) different V genes and (B) antibodies to different antigens are shown. One data point represents one paratope allelic polymorphism. For the boxplot, the middle horizontal line represents the median. The lower and upper hinges represent the first and third quartiles, respectively. The upper whisker extends to the highest data point within a 1.5× inter-quartile range (IQR) of the third quartile, whereas the lower whisker extends to the lowest data point within a 1.5× IQR of the first quartile. Paratope allelic polymorphisms with predicted $\Delta\Delta G > 10$ kcal/mol are shown as 10 kcal/mol. Paratope allelic polymorphisms with predicted $\Delta\Delta G < -5$ kcal/mol are shown as −5 kcal/mol. See also Table S1.

depending on the allele. Among 1,266 IGHV1-69 antibodies from GenBank,[37] 84% were encoded by alleles with Gly50 (Figure 3B). Previous studies have shown that allelic polymorphism V_H G50R would abolish the binding activity of IGHV1-69 antibodies to *S. aureus*.[4] Our BLI experiment further demonstrated that V_H G50R abolished binding of IGHV1-69 antibody 4E10 to the membrane-proximal external region (MPER) of HIV envelope (Env) and reduced binding of another IGHV1-69 antibody, HC84.26.5D, to HCV E2 by around 6-fold (Figure 3A; Figure S2). Of note, 4E10, which was discovered more than 20 years ago,[38] represents a multidonor class of IGHV1-69/IGKV3-20 broadly neutralizing antibodies to HIV.[39] Structural modeling showed that V_H G50R would introduce substantial steric clash between 4E10 and HIV Env (Figure 3C). A similar observation was made for V_H G50R in antibody HC84.26.5D (PDB: 4Z0X)[32] (Figure 3D). A recent preprint has reported that V_H G50R can also abolish the binding activity of another IGHV1-69 antibody to HCV E2,[40] although its epitope differs from that of HC84.26.5D. These findings demonstrate that multiple IGHV1-69 antibodies in the literature with different specificities have a strong preference toward alleles that encode Gly50 rather than Arg50.

Another example was IGLV3-21 residue 50, which has either Asp or Tyr, depending on the allele. Among 347 IGLV3-21 antibodies from GenBank,[37] 66% were encoded by alleles with Asp50 (Figure 3E). Our BLI experiment showed that V_L D50Y abolished the binding activity of IGLV3-21 antibody 5J8 to influenza H1N1 A/California/07/2009 HA and weakened the binding affinity of another IGLV3-21 antibody, Fab234, to *P. falciparum* CSP by around 5-fold (Figure 3A; Figure S2). Structural modeling indicated that V_L D50Y would remove an H-bond and introduce steric clashes between 5J8 and influenza HA (Figure 3F) and, similarly, result in loss of an H-bond between Fab234 and *P. falciparum* CSP (Figure 3G). These observations not only substantiate that a given allelic polymorphism can impact the binding activities of diverse antibodies but also demonstrate that such a phenomenon also arises in light chain.

Importance of minor allelic polymorphisms in broadly neutralizing antibodies

The binding activities of three out of 14 antibodies in our BLI experiment (Figure 3A) were attributed to minor V-gene allelic polymorphisms (i.e., allelic polymorphism frequency <25% among antibodies in GenBank; Table S2). These three antibodies, namely

CellPress
OPEN ACCESS

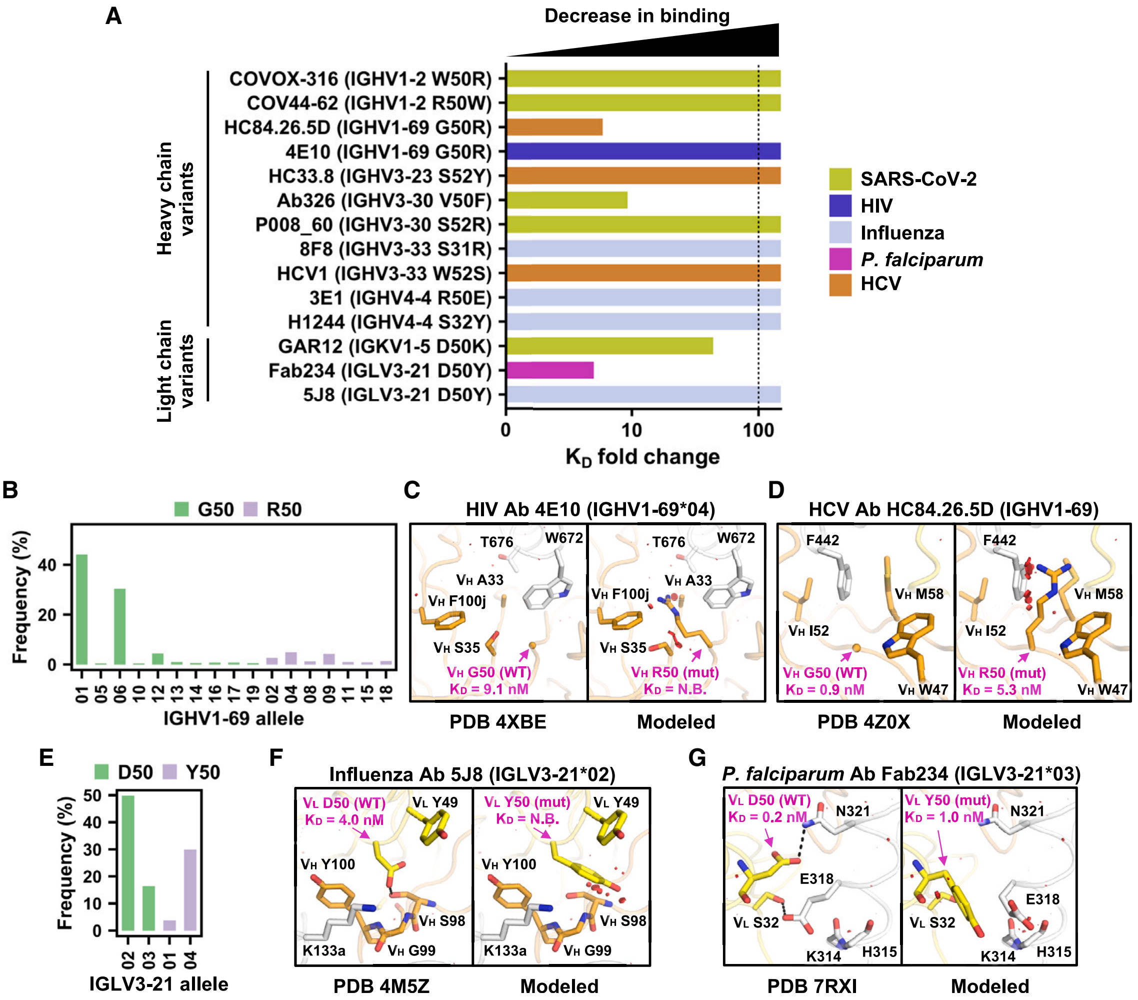

Figure 3. A given allelic polymorphism can impact the binding activity of antibodies to different antigens

(A) The impact of the indicated paratope allelic polymorphisms on the antibody binding affinity of the corresponding antibodies is quantified by the fold change in the binding dissociation constant (K_D). A higher fold change indicates worse binding. Paratope allelic polymorphisms that abolished antibody binding are shown with a fold change of >100. Antibody specificity is color-coded.

(B and E) Allele usages of (B) IGHV1-69 antibodies and (E) IGLV3-21 antibodies are shown. The y axis represents the allele frequency among antibodies in GenBank that are encoded by the indicated V gene. Bar color represents the amino acid identity at the indicated residue position.

(C, D, F, and G) Structural effects of paratope allelic polymorphisms (C) V_H G50R of antibody 4E10 in complex with HIV Env membrane-proximal external region (PDB: 4XBE),[31] (D) V_H G50R of antibody HC84.26.5D in complex with HCV glycoprotein E2 (PDB: 4Z0X),[32] (F) V_L D50Y of antibody 5J8 in complex with influenza H1N1 A/California/07/2009 HA1 subunit (PDB: 4M5Z),[33] and (G) V_L D50Y of Fab234 in complex with C-terminal αTSR domain of *P. falciparum* circumsporozoite protein (PDB: 7RXI)[34] are modeled by FoldX.[21] Left: previously determined experimental structures of antibody-antigen complexes. Right: models of alternative allelic polymorphisms. Allelic polymorphic residues are labeled in magenta. K_D values of wild-type and allelic mutants to antigens were measured by biolayer interferometry (BLI) and are indicated. N.B. represents no binding. Antibody heavy and light chains are shown in orange and yellow, respectively. Antigens are shown in white. Red disks indicate significant van der Waals overlap (distance < 2.8 Å), hence representing a steric clash. H-bonds are represented by black dashed lines. Kabat numbering is applied to all antibodies. Cα atoms of glycines are represented by spheres. The V-gene and allele usage for each antibody is indicated. Of note, the allele usage for HC84.26.5D cannot be assigned unambiguously.

See also Figures S2–S4 and Table S2.

GAR12, COV44-62, and 3E1, are all broadly neutralizing antibodies. GAR12 targets the receptor-binding domain of SARS-CoV-2 spike and was previously shown to neutralize all tested variants of concern, including Omicron BA.1, BA.2, and BA.5.[41] The light chain of GAR12 is encoded by allele *01 of IGKV1-5, which has a minor allelic polymorphism Asp50. Among 744 IGKV1-5 antibodies from GenBank,[37] only 16% were encoded by alleles with Asp50 (allele *01 or *02), whereas the remaining 84% were encoded by allele *03, which had Lys50 (Figure 4A). Our BLI experiment showed that the binding of GAR12 to the receptor-binding domain of SARS-CoV-2 spike was reduced by 44-fold when V_L Asp50 was mutated to the major allelic polymorphism V_L Lys50. Structural modeling indicated that V_L D50K would disrupt an extensive electrostatic interaction network with Arg346 of the receptor-binding domain of SARS-CoV-2 spike and introduce unfavorable electrostatic interactions (Figure 4B).

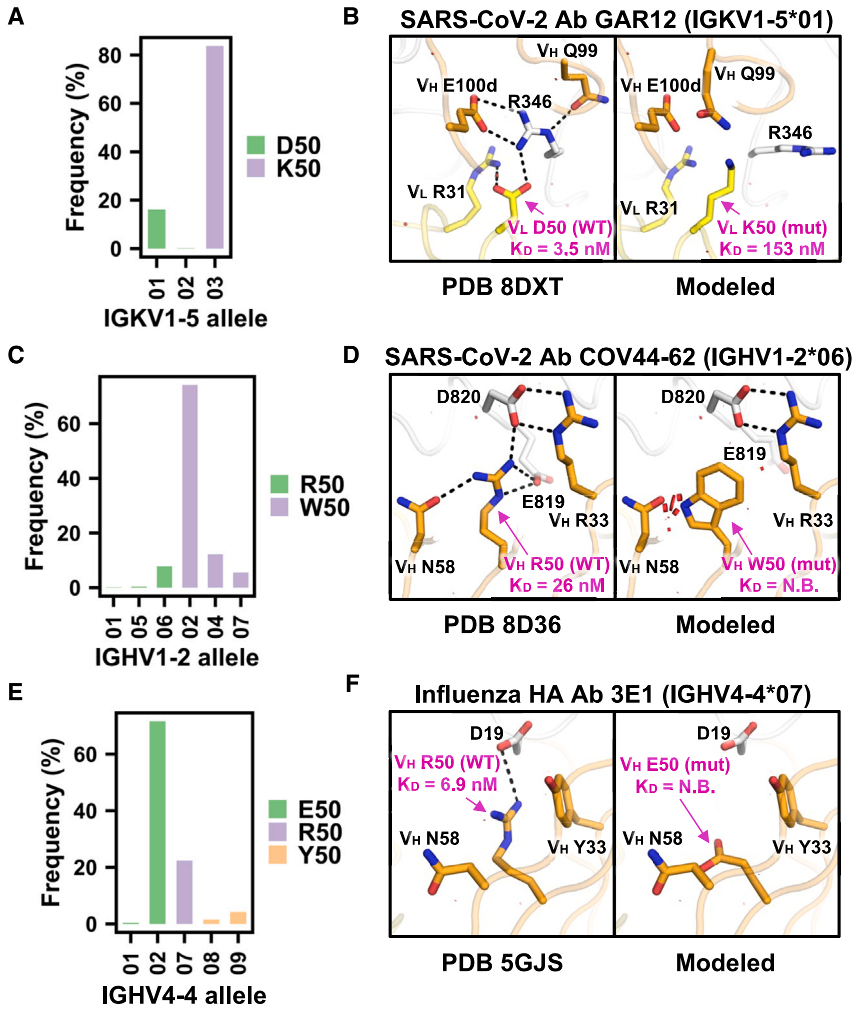

Figure 4. Minor immunoglobulin V-gene allelic polymorphisms are important for the binding activity of diverse antibodies

(A, C, and E) Allele usage of (A) IGKV1-5 antibodies, (C) IGHV1-2 antibodies, and (E) IGHV4-4 antibodies is shown. Bar color represents the amino acid identity at the indicated residue position. The y axis represents the allele frequency among antibodies in GenBank that are encoded by the indicated V gene.

(B, D, and F) Structural effects of paratope allelic polymorphisms (B) V_L D50K of antibody GAR12 in complex with the receptor-binding domain of SARS-CoV-2 spike (PDB: 8DXT),[41] (D) V_H R50W of antibody COV44-62 in complex with the fusion peptide of SARS-CoV-2 spike (PDB: 8D36),[42] and (F) V_H R50E of antibody 3E1 in complex with influenza H1N1 A/California/04/2009 HA (PDB: 5GJS)[43] are modeled by FoldX.[21] Structure visualization has the same style and format as Figure 3, with H-bonds and salt bridges represented by black dashed lines. See also Figures S2–S4 and Table S2.

COV44-62 targets the highly conserved fusion peptide of coronavirus spike and was previously shown to neutralize various coronavirus strains from different genera.[42] The heavy chain of COV44-62 is encoded by allele *06 of IGHV1-2, which has a minor allelic polymorphism Arg50. Among 788 IGHV1-2 antibodies from GenBank,[37] only 8% were encoded by alleles with Arg50, whereas the remaining 92% were encoded by alleles with Trp50 (Figure 4C). Our BLI experiment showed that binding of COV44-62 to the fusion peptide of SARS-CoV-2 spike was abolished by mutating V_H Arg50 to the major allelic polymorphism V_H Trp50 (Figure 3A; Figure S2). Structural modeling indicated that V_H R50W would remove multiple salt bridges between COV44-62 and SARS-CoV-2 fusion peptide as well as introduce steric clashes at the binding interface (Figure 4D).

3E1 targets the conserved stem domain of influenza HA and was previously shown to neutralize influenza A H1 and H5 subtypes.[43] The heavy chain of 3E1 is encoded by IGHV4-4, which has a minor allelic polymorphism Arg50. Among 264 IGHV4-4 antibodies from GenBank,[37] 6%, 22%, and 72% were encoded by alleles with Tyr50, Arg50, and Glu50, respectively (Figure 4E). Our BLI experiment showed that the binding of 3E1 to influenza H1N1 A/California/07/2009 HA was abolished by mutating V_H Arg50 to the major allelic polymorphism V_H Glu50 (Figure 3A; Figure S2). Structural modeling showed that V_H R50E would eliminate a salt bridge between 3E1 and influenza HA (Figure 4F). Overall, these results demonstrate the contribution of minor V-gene allelic polymorphisms to broadly neutralizing antibody responses against different antigens.

DISCUSSION

Previous studies have provided limited examples of how V-gene allelic polymorphisms can affect the binding activity of antibodies of interest.[3–8] By analyzing more than a thousand publicly available antibody-antigen complex structures, our study here shows that the impact of V-gene allelic polymorphisms on antibody binding activity is highly prevalent. Among the 1,048 antibody-antigen complex structures being analyzed, 44% (464/1,048) contain allelic polymorphisms that were predicted to disrupt binding. Besides, the binding activity of all tested antibodies (14 out of 14) was decreased or abolished by alternative allelic polymorphisms. Consistent with these findings, a recent preprint reports that V-gene allelic polymorphisms are linked to the variability of antibody repertoires across individuals.[44] These observations indicate that V-gene allelic polymorphisms can greatly influence antibody responses to different antigens. Consequently, the potency and breadth of antibody response that is elicited by infection or vaccination may be associated with V-gene allele usage. This phenomenon is indeed observed in a phase 1 clinical trial of an HIV vaccine candidate.[8,9] Given that certain broadly neutralizing antibodies rely on minor V-gene allelic polymorphisms, V-gene allelic polymorphisms in the human population are an important consideration in the pursuit of more universal vaccines. At the same time, we

CellPress
OPEN ACCESS

acknowledge that the binding activity of many antibodies are independent of V-gene allelic polymorphisms. Besides, antibodies with different alleles may prefer different epitopes yet ones on the same antigen.[45]

The need, if any, for maintaining multiple alleles of a given V gene in the human population has been previously regarded as an evolutionary mystery.[2] One possibility is that different alleles of a given V gene have different antigen-binding preferences, which would in turn lead to a heterozygote advantage. An example is residue 50 of IGHV1-69, which encodes either Gly or Arg, depending on alleles. V_H G50R abolished the binding activity of IGHV1-69 antibodies to *S. aureus*, as shown in a previous study,[4] as well as HIV Env and HCV E2, as shown in our work here. Nevertheless, a recent study has shown that V_H Arg50 is essential for IGHV1-69 antibodies to interact with the receptor-binding domain of SARS-CoV-2 spike.[3] As a result, mounting an optimal IGHV1-69 antibody response against *S. aureus*, HIV, HCV, and SARS-CoV-2 would require both Gly50 and Arg50 alleles of IGHV1-69. In other words, individuals with either Gly50 or Arg50 in all copies of the IGHV1-69 gene may have difficulties generating an effective IGHV1-69 antibody response against certain pathogens. Another example is residue 50 of IGHV1-2, which encodes either Arg or Trp, depending on alleles. Our work here showed that V_H Arg50 is essential for the binding activity of COV44-62, which is an IGHV1-2 broadly neutralizing coronavirus antibody.[42] In contrast, V_H Trp50 is essential for the binding activity of IGHV1-2 broadly neutralizing HIV antibodies.[8,12,13] Based on our $\Delta\Delta G_{binding}$ prediction results, similar observations can be made for allelic polymorphisms in other germline genes (Figure S3). Nevertheless, future experimental studies will be needed to fully dissect the evolutionary causes and consequences of allelic polymorphisms in different V genes.

In this study, we also identified several FDA-approved therapeutic antibodies where paratope allelic polymorphisms were predicted to strongly disrupt binding to human proteins, including cancer therapeutic targets. These antibodies were identified by phage display screening of human antibody libraries (e.g., avelumab,[46] tralokinumab,[47] and atezolizumab[48]) and immunization of humanized mice (e.g., dupilumab[49] and daratumumab[50]), which are two common methods for antibody discovery. Both methods require cloning of antibody repertoires from human donors. Based on our results, it is likely that the probability of success of antibody discovery through phage display screening and humanized mice depends not only on the antigens but also on the V-gene allelic polymorphisms of the human donors present in the phage-displayed antibody libraries or in the humanized mice. Future antibody discovery may benefit from having multiple human donors with diverse V gene alleles. Given that the market size of monoclonal antibodies continues to grow,[51] understanding the impact of V-gene allelic polymorphisms on antibody discovery will have important public health implications.

While this study indicates that many V-gene allelic polymorphisms can influence antibody binding activity, our analysis only focused on those in the paratope. Previous studies have demonstrated that non-paratope mutations can also affect antibody binding activity.[52–54] Therefore, some non-paratope V-gene allele polymorphisms may possibly do the same. Besides, the diversity of human V-gene alleles is likely higher than what is currently known. Over the past decade, numerous V-gene alleles have been discovered thanks to the advances in computational methods for germline gene inference and long-read third-generation sequencing technologies.[19] The ongoing efforts in understanding the diversity of V genes across human populations and ethnicities, including geographic diversity, will likely reveal many more novel V-gene alleles.[55] Of note, the impact of V-gene allelic polymorphisms on antibody binding are unlikely to be limited to human V genes since a similar observation has been recently reported in IGHV3-73 of rhesus macaque to SARS-CoV-2.[56] Together, it is likely that V-gene allelic polymorphisms, as well as their impact on antibody binding activity, are more widespread than indicated by our and previous studies.[19]

Limitations of the study

For antibodies to pathogens, the existing antibody-antigen complex structures are heavily biased toward those with neutralization activity. It is unclear whether V-gene allelic polymorphisms also have a prevalence effect on the binding activity of non-neutralizing antibodies. In addition, although FoldX has a decent accuracy in identifying destabilizing mutations,[22,23] it only has moderate performance in predicting the magnitude of effect on binding activity.[22] Therefore, caution is needed to infer the change in K_D from the predicted $\Delta\Delta G_{binding}$. Moreover, our study mostly focused on affinity-matured antibodies, including all those in our experimental validation (Figure 3A; Table S2). While we anticipate that the effects of allelic polymorphisms on binding activity should also apply to their corresponding unmutated common ancestor (UCA), experimental confirmation is warranted in future studies. Lastly, this study estimated the frequency of different V-gene alleles using the antibody sequences in GenBank, which came from more than 200 different studies. Although such estimation may deviate from that in the human population due to the bias in sequence deposition, the frequency of different V-gene alleles in various ethnic and geographical backgrounds remains to be comprehended.[55]

STAR★METHODS

Detailed methods are provided in the online version of this paper and include the following:

- KEY RESOURCES TABLE
- RESOURCE AVAILABILITY
  - Lead contact
  - Materials availability
  - Data and code availability
- EXPERIMENTAL MODELS AND SUBJECT DETAILS
  - Cell cultures
- METHOD DETAILS
  - Identification of paratope residues with allelic polymorphisms
  - Predicting the $\Delta\Delta G$ of binding for allelic polymorphisms
  - Germline immunoglobulin V gene and allele assignment of antibodies from GenBank

- ○ Expression and purification of fabs
- ○ Expression and purification of antigens
- ○ Biolayer interferometry (BLI) binding assays
- ● QUANTIFICATION AND STATISTICAL ANALYSIS

SUPPLEMENTAL INFORMATION

Supplemental information can be found online at https://doi.org/10.1016/j.celrep.2023.113194.

ACKNOWLEDGMENTS

We thank Jeanne Matteson and Beverly Ellis for contribution to mammalian cell culture and Wenli Yu, Xueyong Zhu, Re'em Moskovitz, Tossapol Pholcharee, and T.K. Yen Nguyen for assistance in protein production. We are grateful for the SARS-CoV-2 spike (HexaPro) plasmid from Jason McLellan from The University of Texas at Austin. This work was supported by the National Institutes of Health (NIH) R01 AI167910 (N.C.W.), DP2 AT011966 (N.C.W.), and UM1 AI144462 (I.A.W.); the Department of Health and Human Services under contract number 75N93021C00015 (I.A.W. and N.C.W.), the Bill and Melinda Gates Foundation INV-004923 (I.A.W.), and the Searle Scholars Program (N.C.W.).

AUTHOR CONTRIBUTIONS

M.Y. and N.C.W. conceived and designed the study. N.S. and N.C.W. analyzed the antibody structure database and performed mutational stability analysis. M.Y. and N.C.W. performed the structural analysis. M.Y., Z.F., H.L., I.R.S., T.J.C.T., Q.W.T., W.O.O., and L.T. expressed and purified the antibodies and antigens. M.Y. and Z.F. performed the BLI experiment. N.C.W. and I.A.W. provided resources and support. M.Y. and N.C.W. wrote the paper, and all authors reviewed and/or edited the paper.

DECLARATION OF INTERESTS

N.C.W. consults for HeliXon.

Received: June 7, 2023
Revised: September 7, 2023
Accepted: September 14, 2023
Published: September 30, 2023

REFERENCES

1. Briney, B., Inderbitzin, A., Joyce, C., and Burton, D.R. (2019). Commonality despite exceptional diversity in the baseline human antibody repertoire. Nature *566*, 393–397. https://doi.org/10.1038/s41586-019-0879-y.
2. Pennell, M., Rodriguez, O.L., Watson, C.T., and Greiff, V. (2023). The evolutionary and functional significance of germline immunoglobulin gene variation. Trends Immunol. *44*, 7–21. https://doi.org/10.1016/j.it.2022.11.001.
3. Pushparaj, P., Nicoletto, A., Sheward, D.J., Das, H., Castro Dopico, X., Perez Vidakovics, L., Hanke, L., Chernyshev, M., Narang, S., Kim, S., et al. (2023). Immunoglobulin germline gene polymorphisms influence the function of SARS-CoV-2 neutralizing antibodies. Immunity *56*, 193–206.e7. https://doi.org/10.1016/j.immuni.2022.12.005.
4. Yeung, Y.A., Foletti, D., Deng, X., Abdiche, Y., Strop, P., Glanville, J., Pitts, S., Lindquist, K., Sundar, P.D., Sirota, M., et al. (2016). Germline-encoded neutralization of a Staphylococcus aureus virulence factor by the human antibody repertoire. Nat. Commun. *7*, 13376. https://doi.org/10.1038/ncomms13376.
5. Imkeller, K., Scally, S.W., Bosch, A., Martí, G.P., Costa, G., Triller, G., Murugan, R., Renna, V., Jumaa, H., Kremsner, P.G., et al. (2018). Antihomotypic affinity maturation improves human B cell responses against a repetitive epitope. Science *360*, 1358–1362. https://doi.org/10.1126/science.aar5304.
6. Yuan, M., Wang, Y., Lv, H., Tan, T.J.C., Wilson, I.A., and Wu, N.C. (2022). Molecular analysis of a public cross-neutralizing antibody response to SARS-CoV-2. Cell Rep. *41*, 111650. https://doi.org/10.1016/j.celrep.2022.111650.
7. Alam, S.M., Liao, H.X., Dennison, S.M., Jaeger, F., Parks, R., Anasti, K., Foulger, A., Donathan, M., Lucas, J., Verkoczy, L., et al. (2011). Differential reactivity of germ line allelic variants of a broadly neutralizing HIV-1 antibody to a gp41 fusion intermediate conformation. J. Virol. *85*, 11725–11731. https://doi.org/10.1128/JVI.05680-11.
8. deCamp, A.C., Corcoran, M.M., Fulp, W.J., Willis, J.R., Cottrell, C.A., Bader, D.L., Kalyuzhniy, O., Leggat, D.J., Cohen, K.W., Hyrien, O., et al. (2023). Human immunoglobulin gene allelic variation impacts germline-targeting vaccine priming. Preprint at medRxiv, 2023.03.10.23287126. https://doi.org/10.1101/2023.03.10.23287126.
9. Lee, J.H., Toy, L., Kos, J.T., Safonova, Y., Schief, W.R., Havenar-Daughton, C., Watson, C.T., and Crotty, S. (2021). Vaccine genetics of IGHV1-2 VRC01-class broadly neutralizing antibody precursor naive human B cells. NPJ Vaccines *6*, 113. https://doi.org/10.1038/s41541-021-00376-7.
10. Sangesland, M., Torrents de la Peña, A., Boyoglu-Barnum, S., Ronsard, L., Mohamed, F.A.N., Moreno, T.B., Barnes, R.M., Rohrer, D., Lonberg, N., Ghebremichael, M., et al. (2022). Allelic polymorphism controls autoreactivity and vaccine elicitation of human broadly neutralizing antibodies against influenza virus. Immunity *55*, 1693–1709.e8. https://doi.org/10.1016/j.immuni.2022.07.006.
11. Leggat, D.J., Cohen, K.W., Willis, J.R., Fulp, W.J., deCamp, A.C., Kalyuzhniy, O., Cottrell, C.A., Menis, S., Finak, G., Ballweber-Fleming, L., et al. (2022). Vaccination induces HIV broadly neutralizing antibody precursors in humans. Science *378*, eadd6502. https://doi.org/10.1126/science.add6502.
12. Jardine, J., Julien, J.P., Menis, S., Ota, T., Kalyuzhniy, O., McGuire, A., Sok, D., Huang, P.S., MacPherson, S., Jones, M., et al. (2013). Rational HIV immunogen design to target specific germline B cell receptors. Science *340*, 711–716. https://doi.org/10.1126/science.1234150.
13. West, A.P., Jr., Diskin, R., Nussenzweig, M.C., and Bjorkman, P.J. (2012). Structural basis for germ-line gene usage of a potent class of antibodies targeting the CD4-binding site of HIV-1 gp120. Proc. Natl. Acad. Sci. USA. *109*, E2083–E2090. https://doi.org/10.1073/pnas.1208984109.
14. Avnir, Y., Watson, C.T., Glanville, J., Peterson, E.C., Tallarico, A.S., Bennett, A.S., Qin, K., Fu, Y., Huang, C.Y., Beigel, J.H., et al. (2016). IGHV1-69 polymorphism modulates anti-influenza antibody repertoires, correlates with IGHV utilization shifts and varies by ethnicity. Sci. Rep. *6*, 20842. https://doi.org/10.1038/srep20842.
15. Pappas, L., Foglierini, M., Piccoli, L., Kallewaard, N.L., Turrini, F., Silacci, C., Fernandez-Rodriguez, B., Agatic, G., Giacchetto-Sasselli, I., Pellicciotta, G., et al. (2014). Rapid development of broadly influenza neutralizing antibodies through redundant mutations. Nature *516*, 418–422. https://doi.org/10.1038/nature13764.
16. Throsby, M., van den Brink, E., Jongeneelen, M., Poon, L.L.M., Alard, P., Cornelissen, L., Bakker, A., Cox, F., van Deventer, E., Guan, Y., et al. (2008). Heterosubtypic neutralizing monoclonal antibodies cross-protective against H5N1 and H1N1 recovered from human IgM+ memory B cells. PLoS One *3*, e3942. https://doi.org/10.1371/journal.pone.0003942.
17. Johnson, T.A., Mashimo, Y., Wu, J.Y., Yoon, D., Hata, A., Kubo, M., Takahashi, A., Tsunoda, T., Ozaki, K., Tanaka, T., et al. (2021). Association of an IGHV3-66 gene variant with Kawasaki disease. J. Hum. Genet. *66*, 475–489. https://doi.org/10.1038/s10038-020-00864-z.
18. Parks, T., Mirabel, M.M., Kado, J., Auckland, K., Nowak, J., Rautanen, A., Mentzer, A.J., Marijon, E., Jouven, X., Perman, M.L., et al. (2017). Association between a common immunoglobulin heavy chain allele and rheumatic heart disease risk in Oceania. Nat. Commun. *8*, 14946. https://doi.org/10.1038/ncomms14946.

CellPress
OPEN ACCESS

19. Dopico, X.C., Mandolesi, M., and Hedestam, G.B.K. (2023). Untangling immunoglobulin genotype-function associations. Immunol. Lett. https://doi.org/10.1016/j.imlet.2023.05.003.

20. Dunbar, J., Krawczyk, K., Leem, J., Baker, T., Fuchs, A., Georges, G., Shi, J., and Deane, C.M. (2014). SAbDab: the structural antibody database. Nucleic Acids Res. *42*, D1140–D1146. https://doi.org/10.1093/nar/gkt1043.

21. Delgado, J., Radusky, L.G., Cianferoni, D., and Serrano, L. (2019). FoldX 5.0: working with RNA, small molecules and a new graphical interface. Bioinformatics *35*, 4168–4169. https://doi.org/10.1093/bioinformatics/btz184.

22. Potapov, V., Cohen, M., and Schreiber, G. (2009). Assessing computational methods for predicting protein stability upon mutation: good on average but not in the details. Protein Eng. Des. Sel. *22*, 553–560. https://doi.org/10.1093/protein/gzp030.

23. Buß, O., Rudat, J., and Ochsenreither, K. (2018). FoldX as protein engineering tool: better than random based approaches? Comput. Struct. Biotechnol. J. *16*, 25–33. https://doi.org/10.1016/j.csbj.2018.01.002.

24. Lefranc, M.P., Giudicelli, V., Ginestoux, C., Jabado-Michaloud, J., Folch, G., Bellahcene, F., Wu, Y., Gemrot, E., Brochet, X., Lane, J., et al. (2009). IMGT, the international ImMunoGeneTics information system. Nucleic Acids Res. *37*, D1006–D1012. https://doi.org/10.1093/nar/gkn838.

25. Liu, K., Tan, S., Chai, Y., Chen, D., Song, H., Zhang, C.W.H., Shi, Y., Liu, J., Tan, W., Lyu, J., et al. (2017). Structural basis of anti-PD-L1 monoclonal antibody avelumab for tumor therapy. Cell Res. *27*, 151–153. https://doi.org/10.1038/cr.2016.102.

26. Lieu, R., Antonysamy, S., Druzina, Z., Ho, C., Kang, N.R., Pustilnik, A., Wang, J., and Atwell, S. (2020). Rapid and robust antibody Fab fragment crystallization utilizing edge-to-edge beta-sheet packing. PLoS One *15*, e0232311. https://doi.org/10.1371/journal.pone.0232311.

27. Popovic, B., Breed, J., Rees, D.G., Gardener, M.J., Vinall, L.M.K., Kemp, B., Spooner, J., Keen, J., Minter, R., Uddin, F., et al. (2017). Structural characterisation reveals mechanism of IL-13-neutralising monoclonal antibody tralokinumab as inhibition of binding to IL-13Rα1 and IL-13Rα2. J. Mol. Biol. *429*, 208–219. https://doi.org/10.1016/j.jmb.2016.12.005.

28. Zhang, F., Qi, X., Wang, X., Wei, D., Wu, J., Feng, L., Cai, H., Wang, Y., Zeng, N., Xu, T., et al. (2017). Structural basis of the therapeutic anti-PD-L1 antibody atezolizumab. Oncotarget *8*, 90215–90224. https://doi.org/10.18632/oncotarget.21652.

29. Park, U.B., Jeong, T.J., Gu, N., Lee, H.T., and Heo, Y.S. (2022). Molecular basis of PD-1 blockade by dostarlimab, the FDA-approved antibody for cancer immunotherapy. Biochem. Biophys. Res. Commun. *599*, 31–37. https://doi.org/10.1016/j.bbrc.2022.02.026.

30. Lee, H.T., Kim, Y., Park, U.B., Jeong, T.J., Lee, S.H., and Heo, Y.S. (2021). Crystal structure of CD38 in complex with daratumumab, a first-in-class anti-CD38 antibody drug for treating multiple myeloma. Biochem. Biophys. Res. Commun. *536*, 26–31. https://doi.org/10.1016/j.bbrc.2020.12.048.

31. Irimia, A., Sarkar, A., Stanfield, R.L., and Wilson, I.A. (2016). Crystallographic identification of lipid as an integral component of the epitope of HIV broadly neutralizing antibody 4E10. Immunity *44*, 21–31. https://doi.org/10.1016/j.immuni.2015.12.001.

32. Keck, Z.Y., Wang, Y., Lau, P., Lund, G., Rangarajan, S., Fauvelle, C., Liao, G.C., Holtsberg, F.W., Warfield, K.L., Aman, M.J., et al. (2016). Affinity maturation of a broadly neutralizing human monoclonal antibody that prevents acute hepatitis C virus infection in mice. Hepatology *64*, 1922–1933. https://doi.org/10.1002/hep.28850.

33. Hong, M., Lee, P.S., Hoffman, R.M.B., Zhu, X., Krause, J.C., Laursen, N.S., Yoon, S.I., Song, L., Tussey, L., Crowe, J.E., Jr., et al. (2013). Antibody recognition of the pandemic H1N1 Influenza virus hemagglutinin receptor binding site. J. Virol. *87*, 12471–12480. https://doi.org/10.1128/JVI.01388-13.

34. Beutler, N., Pholcharee, T., Oyen, D., Flores-Garcia, Y., MacGill, R.S., Garcia, E., Calla, J., Parren, M., Yang, L., Volkmuth, W., et al. (2022). A novel CSP C-terminal epitope targeted by an antibody with protective activity against *Plasmodium falciparum*. PLoS Pathog. *18*, e1010409. https://doi.org/10.1371/journal.ppat.1010409.

35. Chen, F., Tzarum, N., Wilson, I.A., and Law, M. (2019). V_H1-69 antiviral broadly neutralizing antibodies: genetics, structures, and relevance to rational vaccine design. Curr. Opin. Virol. *34*, 149–159. https://doi.org/10.1016/j.coviro.2019.02.004.

36. Lerner, R.A. (2011). Rare antibodies from combinatorial libraries suggests an S.O.S. component of the human immunological repertoire. Mol. Biosyst. *7*, 1004–1012. https://doi.org/10.1039/c0mb00310g.

37. Benson, D.A., Cavanaugh, M., Clark, K., Karsch-Mizrachi, I., Lipman, D.J., Ostell, J., and Sayers, E.W. (2013). Nucleic Acids Res. *41*, D36–D42. https://doi.org/10.1093/nar/gks1195.

38. Zwick, M.B., Labrijn, A.F., Wang, M., Spenlehauer, C., Saphire, E.O., Binley, J.M., Moore, J.P., Stiegler, G., Katinger, H., Burton, D.R., and Parren, P.W. (2001). Broadly neutralizing antibodies targeted to the membrane-proximal external region of human immunodeficiency virus type 1 glycoprotein gp41. J. Virol. *75*, 10892–10905. https://doi.org/10.1128/JVI.75.22.10892-10905.2001.

39. Krebs, S.J., Kwon, Y.D., Schramm, C.A., Law, W.H., Donofrio, G., Zhou, K.H., Gift, S., Dussupt, V., Georgiev, I.S., Schätzle, S., et al. (2019). Longitudinal analysis reveals early development of three MPER-directed neutralizing antibody lineages from an HIV-1-infected individual. Immunity *50*, 677–691.e13. https://doi.org/10.1016/j.immuni.2019.02.008.

40. Capella-Pujol, J., de Gast, M., Radić, L., Zon, I., Chumbe, A., Koekkoek, S., Olijhoek, W., Schinkel, J., Gils, M.J.v., Sanders, R., and Sliepen, K. (2023). Signatures of V_H1-69-derived hepatitis C virus neutralizing antibody precursors defined by binding to envelope glycoproteins. Nat. Commun. *14*, 4036. https://doi.org/10.1038/s41467-023-39690-0.

41. Rouet, R., Henry, J.Y., Johansen, M.D., Sobti, M., Balachandran, H., Langley, D.B., Walker, G.J., Lenthall, H., Jackson, J., Ubiparipovic, S., et al. (2023). Broadly neutralizing SARS-CoV-2 antibodies through epitope-based selection from convalescent patients. Nat. Commun. *14*, 687. https://doi.org/10.1038/s41467-023-36295-5.

42. Dacon, C., Tucker, C., Peng, L., Lee, C.C.D., Lin, T.H., Yuan, M., Cong, Y., Wang, L., Purser, L., Williams, J.K., et al. (2022). Broadly neutralizing antibodies target the coronavirus fusion peptide. Science *377*, 728–735. https://doi.org/10.1126/science.abq3773.

43. Wang, W., Sun, X., Li, Y., Su, J., Ling, Z., Zhang, T., Wang, F., Zhang, H., Chen, H., Ding, J., and Sun, B. (2016). Human antibody 3E1 targets the HA stem region of H1N1 and H5N6 influenza A viruses. Nat. Commun. *7*, 13577. https://doi.org/10.1038/ncomms13577.

44. Rodriguez, O.L., Safonova, Y., Silver, C.A., Shields, K., Gibson, W.S., Kos, J.T., Tieri, D., Ke, H., Jackson, K.J.L., Boyd, S.D., et al. (2023). Genetic variation in the immunoglobulin heavy chain locus shapes the human antibody repertoire. Nat. Commun. *14*, 4419. https://doi.org/10.1038/s41467-023-40070-x.

45. Bennett, M.R., Dong, J., Bombardi, R.G., Soto, C., Parrington, H.M., Nargi, R.S., Schoeder, C.T., Nagel, M.B., Schey, K.L., Meiler, J., et al. (2019). Human V_H1-69 gene-encoded human monoclonal antibodies against *Staphylococcus aureus* IsdB use at least three distinct modes of binding to inhibit bacterial growth and pathogenesis. mBio *10*, e02473-19. https://doi.org/10.1128/mBio.02473-19.

46. Nastri, H.G., Iffland, C., Leger, O., An, Q., Cartwright, M., Mckenna, S.D., Sood, V.D., and Hao, G. (2012). Anti-PD-L1 antibodies and uses thereof. In Patent WO2013079174A1.

47. Monk, P., Jermutus, L., Shorrock, C., and Minter, R. (2004). Human Antibody Molecules for IL-13. In Patent US20070128192A1.

48. Santini, F.C., and Rudin, C.M. (2017). Atezolizumab for the treatment of non-small cell lung cancer. Expert Rev. Clin. Pharmacol. *10*, 935–945. https://doi.org/10.1080/17512433.2017.1356717.

49. Martin, J.H., Huang, T.T., Fairhurst, J.L., and Papadopoulos, N.J. (2008). High affinity human antibodies to human IL-4 receptor. In Patent US7608693B2.

50. de Weers, M., Tai, Y.T., van der Veer, M.S., Bakker, J.M., Vink, T., Jacobs, D.C.H., Oomen, L.A., Peipp, M., Valerius, T., Slootstra, J.W., et al. (2011). Daratumumab, a novel therapeutic human CD38 monoclonal antibody, induces killing of multiple myeloma and other hematological tumors. J. Immunol. *186*, 1840–1848. https://doi.org/10.4049/jimmunol.1003032.

51. Grilo, A.L., and Mantalaris, A. (2019). The increasingly human and profitable monoclonal antibody market. Trends Biotechnol. *37*, 9–16. https://doi.org/10.1016/j.tibtech.2018.05.014.

52. Foote, J., and Winter, G. (1992). Antibody framework residues affecting the conformation of the hypervariable loops. J. Mol. Biol. *224*, 487–499. https://doi.org/10.1016/0022-2836(92)91010-m.

53. Wu, N.C., Andrews, S.F., Raab, J.E., O'Connell, S., Schramm, C.A., Ding, X., Chambers, M.J., Leung, K., Wang, L., Zhang, Y., et al. (2020). Convergent evolution in breadth of two V_H6-1-encoded influenza antibody clonotypes from a single donor. Cell Host Microbe *28*, 434–444.e4. https://doi.org/10.1016/j.chom.2020.06.003.

54. Klein, F., Diskin, R., Scheid, J.F., Gaebler, C., Mouquet, H., Georgiev, I.S., Pancera, M., Zhou, T., Incesu, R.B., Fu, B.Z., et al. (2013). Somatic mutations of the immunoglobulin framework are generally required for broad and potent HIV-1 neutralization. Cell *153*, 126–138. https://doi.org/10.1016/j.cell.2013.03.018.

55. Peng, K., Safonova, Y., Shugay, M., Popejoy, A.B., Rodriguez, O.L., Breden, F., Brodin, P., Burkhardt, A.M., Bustamante, C., Cao-Lormeau, V.M., et al. (2021). Diversity in immunogenomics: the value and the challenge. Nat. Methods *18*, 588–591. https://doi.org/10.1038/s41592-021-01169-5.

56. Feng, Y., Yuan, M., Powers, J.M., Hu, M., Munt, J.E., Arunachalam, P.S., Leist, S.R., Bellusci, L., Kim, J., Sprouse, K.R., et al. (2023). Broadly neutralizing antibodies against sarbecoviruses generated by immunization of macaques with an AS03-adjuvanted COVID-19 vaccine. Sci. Transl. Med. *15*, eadg7404. https://doi.org/10.1126/scitranslmed.adg7404.

57. Ekiert, D.C., Kashyap, A.K., Steel, J., Rubrum, A., Bhabha, G., Khayat, R., Lee, J.H., Dillon, M.A., O'Neil, R.E., Faynboym, A.M., et al. (2012). Cross-neutralization of influenza A viruses mediated by a single antibody loop. Nature *489*, 526–532. https://doi.org/10.1038/nature11414.

58. Kabsch, W., and Sander, C. (1983). Dictionary of protein secondary structure: pattern recognition of hydrogen-bonded and geometrical features. Biopolymers *22*, 2577–2637. https://doi.org/10.1002/bip.360221211.

59. Dunbar, J., and Deane, C.M. (2016). ANARCI: antigen receptor numbering and receptor classification. Bioinformatics *32*, 298–300. https://doi.org/10.1093/bioinformatics/btv552.

60. Soto, C., Finn, J.A., Willis, J.R., Day, S.B., Sinkovits, R.S., Jones, T., Schmitz, S., Meiler, J., Branchizio, A., and Crowe, J.E., Jr. (2020). PyIR: a scalable wrapper for processing billions of immunoglobulin and T cell receptor sequences using IgBLAST. BMC Bioinf. *21*, 314. https://doi.org/10.1186/s12859-020-03649-5.

61. Ye, J., Ma, N., Madden, T.L., and Ostell, J.M. (2013). IgBLAST: an immunoglobulin variable domain sequence analysis tool. Nucleic Acids Res. *41*, W34–W40. https://doi.org/10.1093/nar/gkt382.

62. Yuan, M., Zhu, X., He, W.T., Zhou, P., Kaku, C.I., Capozzola, T., Zhu, C.Y., Yu, X., Liu, H., Yu, W., et al. (2022). A broad and potent neutralization epitope in SARS-related coronaviruses. Proc. Natl. Acad. Sci. USA. *119*, e2205784119. https://doi.org/10.1073/pnas.2205784119.

63. Hsieh, C.L., Goldsmith, J.A., Schaub, J.M., DiVenere, A.M., Kuo, H.C., Javanmardi, K., Le, K.C., Wrapp, D., Lee, A.G., Liu, Y., et al. (2020). Structure-based design of prefusion-stabilized SARS-CoV-2 spikes. Science *369*, 1501–1505. https://doi.org/10.1126/science.abd0826.

64. Kong, L., Giang, E., Nieusma, T., Kadam, R.U., Cogburn, K.E., Hua, Y., Dai, X., Stanfield, R.L., Burton, D.R., Ward, A.B., et al. (2013). Hepatitis C virus E2 envelope glycoprotein core structure. Science *342*, 1090–1094. https://doi.org/10.1126/science.1243876.

STAR★METHODS

KEY RESOURCES TABLE

REAGENT or RESOURCE	SOURCE	IDENTIFIER
Chemicals, peptides, and recombinant proteins		
NEBuilder HiFi DNA Assembly Master Mix	New England Biolabs	Cat#E2621L
FuGENE HD Transfection Reagent	FuGENE	#E231A
ExpiCHO Expression System Kit	Gibco	#A29133
Expi293 Expression System Kit	Gibco	#A14635
SARS-CoV-2 spike and RBD proteins	In house	N/A
H1N1 A/California/07/2009 HA protein	In house	N/A
H1N1 A/Beijing/262/1995 HA protein	In house	N/A
H2N2 A/Japan/305/1957 HA protein	In house	N/A
HCV (isolate H77) E2 domain protein	In house	N/A
HIV Env MPER peptide	GenScript	N/A
SARS-CoV-2 fusion peptide	GenScript	N/A
Opti-MEM Reduced Serum Medium	Thermo Fisher Scientific	Cat#31985070
FreeStyle 293 expression medium	GIBCO	Cat#12338002
Insect-XPRESS Protein-free Insect Cell Medium with L-glutamine	Lonza	Cat#BP12-730Q
PEI MAX transfection reagent	Polysciences	Cat#24765-1
SARS-CoV-2 fusion peptide	GenScript	N/A
Critical commercial assays		
Octet NTA Biosensors	Sartorius	Cat#18-5101
Octet Streptavidin (SA) Biosensor	Sartorius	Cat#18-5019
Octet Anti-Human Fab-CH1 2ND Generation (FAB2G) Biosensors	Sartorius	Cat#18-5125
Experimental models: Cell lines		
ExpiCHO cells	Thermo Fisher Scientific	Cat#A29127; RRID: CVCL_5J31
Expi293F cells	Thermo Fisher Scientific	Cat#A14527; RRID: CVCL_D615
FreeStyle HEK293F cells	Gibco	Cat#R79007
Sf9 cells	ATCC	RRID: CVCL_0549
High Five Cells	Thermo Fisher Scientific	RRID: CVCL_C190
Recombinant DNA		
phCMV3	Genlantis	Cat#P003300
pFastBac	Ekiert et al.[57]	N/A
Software and algorithms		
Python	https://www.python.org/	N/A
R	https://www.r-project.org/	N/A
V-Quest online tool	IMGT	https://imgt.org/
FoldX	FoldX Suite	https://foldxsuite.crg.eu/
PyMOL	PyMOL by Schrödinger	https://pymol.org
Custom scripts	This study	https://doi.org/10.5281/zenodo.8330193
Other		
0.2 µm membrane filters	Fisher Scientific	Cat#564-0020
HisPur Ni-NTA Resin	Thermo Fisher Scientific	Cat#88221
CaptureSelect CH1-XL Pre-packed Column	Thermo Fisher Scientific	Cat#494346201
Superdex 200 Increase10/300 GL column	GE Healthcare	Cat#GE28-9909-44

(*Continued on next page*)

Continued

REAGENT or RESOURCE	SOURCE	IDENTIFIER
Amicon tubes (100K, 30K, 10K)	Millipore	Cat#UFC9100, Cat#UFC9030, Cat#UFC9010
Bio-One Polypropylene 96-well F-Bottom Microplates	Greiner	Cat#655209

RESOURCE AVAILABILITY

Lead contact

Information and requests for resources should be directed to and will be fulfilled by the lead contact, Nicholas C. Wu (nicwu@illinois.edu).

Materials availability

All plasmids generated in this study are available from the lead contact without restriction.

Data and code availability

- Information on the 1,150 paratope allelic polymorphisms that were analyzed in this study are in Table S1.
- Custom python scripts for analyzing the deep mutational scanning data have been deposited to https://doi.org/10.5281/zenodo.8330193.
- Any additional information required to reanalyze the data reported in this paper is available from the lead contact upon request.

EXPERIMENTAL MODELS AND SUBJECT DETAILS

Cell cultures

FreeStyle HEK293F cells (human embryonic kidney cells, female), ExpiCHO cells (Chinese hamster ovary cells, female) and Expi293F cells (human embryonic kidney cells, female) were maintained in FreeStyle 293 expression medium, ExpiCHO expression medium, and Expi293 expression medium, respectively, at 37°C with 8% CO_2 according to the manufacturer's instructions (Thermo Fisher Scientific). Sf9 cells (*Spodoptera frugiperda* ovarian cells, female) and High Five cells (*Trichoplusia ni* ovarian cells, female) were maintained in Insect-XPRESS medium (Lonza).

METHOD DETAILS

Identification of paratope residues with allelic polymorphisms

A total of 3,240 human antibody-antigen complex structures were downloaded as PDB files from the Structural Antibody Database (SAbDab, http://opig.stats.ox.ac.uk/webapps/sabdab).[20] Next, PDB files with more than one antibody were filtered out, leaving 1,416 complex structures. We further discarded the following PDB files due to formatting issues: 7T1W, 7T1X, 6TUL, 6SS4, 6SS5, 7DWT, 7DWU, 6SS2, 6ZJG, 7T0W, 6YXM, 6TKF, 6TKE, 6TKD, 6TKC, 3J6U, 7R8U, and 6YXL, leaving 1,048 complex structures. For each PDB file, an apo antibody structure was generated by removing the antigen from the PDB file. Relative solvent accessibility (RSA) for each antibody residue, either in apo form or in complex with antigen, was computed by DSSP.[58] Residues with a higher RSA value in the apo antibody structure than the complex structure (i.e., $RSA_{apo\ antibody} - RSA_{complex} > 0$) were defined as paratope residues. Germline sequences for human IGHV and IGK(L)V genes were downloaded from the IMGT database (https://www.imgt.org/)[24] on November 25, 2021. Each position of the antibody sequences in the PDB files and the germline immunoglobulin V gene sequences was numbered according to Kabat numbering using ANARCI.[59]

The germline V genes of each antibody were identified using PyIR,[60] a wrapper for the IgBLAST.[61] For each paratope residue, its allelic polymorphisms, if any, across different alleles of the corresponding germline V gene were compared. Paratope residues with no allelic polymorphism were excluded from downstream analysis. In addition, if the amino acid sequence of a given paratope residue was not germline-encoded, such a paratope residue was also discarded. If a paratope residue has two alternative allelic polymorphisms, both were investigated. In summary, a total of 1,150 paratope allelic polymorphisms across 544 antibody-antigen complex structures were identified. Of note, alleles were not reported for antibodies with ambiguity in allele assignment (e.g., HC84.26.5D in Figure 3D).

Predicting the ΔΔG of binding for allelic polymorphisms

The ΔΔG for each paratope allelic polymorphisms was predicted using FoldX.[21] For each paratope allelic polymorphism of a given antibody, two ΔΔG values were predicted, one for the apo antibody structure ($\Delta\Delta G_{apo\ antibody}$) and the other for the antibody-antigen complex structure ($\Delta\Delta G_{complex}$). Apo antibody structures were generated by extracting the antibody coordinates from the PDB files. Predicted ΔΔG of antibody-antigen binding ($\Delta\Delta G_{binding}$) was computed as:

$$\text{Predicted } \Delta\Delta G_{binding} = \text{Predicted } \Delta\Delta G_{complex} - \text{Predicted } \Delta\Delta G_{apo\ antibody}$$

Germline immunoglobulin V gene and allele assignment of antibodies from GenBank

The germline immunoglobulin V genes and alleles of 12,487 antibodies from GenBank (www.ncbi.nlm.nih.gov/genbank)[37] were identified using PyIR.[60] Antibodies with ambiguity in allele assignment were excluded from our analysis.

Expression and purification of fabs

The heavy and light chains of Fabs were cloned into phCMV3 vector. PCR-based mutagenesis was performed to generate the alternative allelic polymorphisms. The plasmids were transiently co-transfected into ExpiCHO cells at a ratio of 2:1 (heavy chain to light chain) using ExpiFectamine CHO Reagent (Thermo Fisher Scientific) according to the manufacturer's instructions. The supernatant was collected at 7 days post-transfection. The Fab was purified with a CaptureSelect CH1-XL Pre-packed Column (Thermo Fisher Scientific), followed by a buffer exchange to Dulbecco's Phosphate Buffered Saline (PBS, pH 7.4).

Expression and purification of antigens

SARS-CoV-2 receptor-binding domain was expressed in High Five cells and purified with Ni-NTA resin followed by size exclusion as described previously.[62] SARS-CoV-2 whole spike protein HexaPro was a gift from Jason McLellan (Addgene plasmid # 154754). SARS-CoV-2 HexaPro was expressed in Expi293F cells and purified with Ni-NTA resin followed by size exclusion as described previously.[63] The influenza hemagglutinin (HA) proteins from H1N1 A/California/07/2009, H1N1 A/Beijing/262/1995, and H2N2 A/Japan/305/1957 were expressed in High Five cells, purified with Ni-NTA resin followed by size exclusion, and biotinylated as described previously.[57] The HCV (isolate H77) E2 domain was expressed in HEK293S cells and purified as described previously.[64] The biotinylated SARS-CoV-2 fusion peptide (N′-biotin-DPSKPSKRSFIEDLLFNKVT-C′) and His-tagged HIV Env MPER peptide (N′-NWF DITNWLWYIKSGGSHHHHHHHH-C′) were chemically synthesized by GenScript.

Biolayer interferometry (BLI) binding assays

Binding assays were performed by biolayer interferometry (BLI) using an Octet Red instrument (FortéBio). 20 μg/mL of antibodies, antigens, or peptides in 1x kinetics buffer (1x PBS, pH 7.4, 0.01% BSA and 0.002% Tween 20) were loaded onto different types of sensors, and then incubated with 33 nM, 100 nM, and 300 nM of binders. All antibodies were in Fab format. Specifically, biotinylated SARS-CoV-2 fusion peptide was loaded onto Streptavidin (SA) sensors and incubated with COV44-62 (WT or V_H R50W). Biotinylated αTSR domain of *P. falciparum* was loaded onto SA sensors and incubated with Fab234 (WT or V_L D50Y). Biotinylated influenza HA from H1N1 A/California/07/2009 was loaded onto SA sensors and incubated with 3E1 (WT or V_H R50E), or 5J8 (WT or V_L D50). Biotinylated influenza HA from H1N1 A/Beijing/262/1995 was loaded onto SA sensors and incubated with H1244 (WT or V_H S32Y). Biotinylated virus HA from H2N2 A/Japan/305/1957 was loaded onto SA sensors and incubated with 8F8 (WT or V_H S31R). His_6-tagged SARS-CoV-2 receptor-binding domain was loaded onto Ni-NTA sensors and incubated with GAR12 (WT or V_L D50K), Ab326 (WT or V_H V50F), or COVOX-316 (WT or V_H W50R). His-tagged HIV Env MPER peptide was loaded onto Ni-NTA sensors and incubated with 4E10 (WT or V_H G50R). HC33.8 (WT or V_H S52Y), HC84.26.5D (WT or V_H G50R), or HCV1 (WT or V_H W52S) was loaded onto Anti-Human Fab-CH1 2nd Generation (FAB2G) sensors and incubated with HCV E2. P008_60 (WT or V_H S52R) was loaded onto FAB2G sensors and incubated with SARS-CoV-2 spike protein.[63] The assay consisted of five steps: 1) baseline; 2) loading; 3) baseline; 4) association; and 5) dissociation. For estimating the K_D values, a 1:1 binding model was used.

QUANTIFICATION AND STATISTICAL ANALYSIS

Standard deviation for K_D estimation was computed by Octet analysis software 9.0. Spearman's rank correlation coefficient was computed in R.

Cell Reports, Volume *42*

Supplemental information

Widespread impact of immunoglobulin V-gene allelic polymorphisms on antibody reactivity

Meng Yuan, Ziqi Feng, Huibin Lv, Natalie So, Ivana R. Shen, Timothy J.C. Tan, Qi Wen Teo, Wenhao O. Ouyang, Logan Talmage, Ian A. Wilson, and Nicholas C. Wu

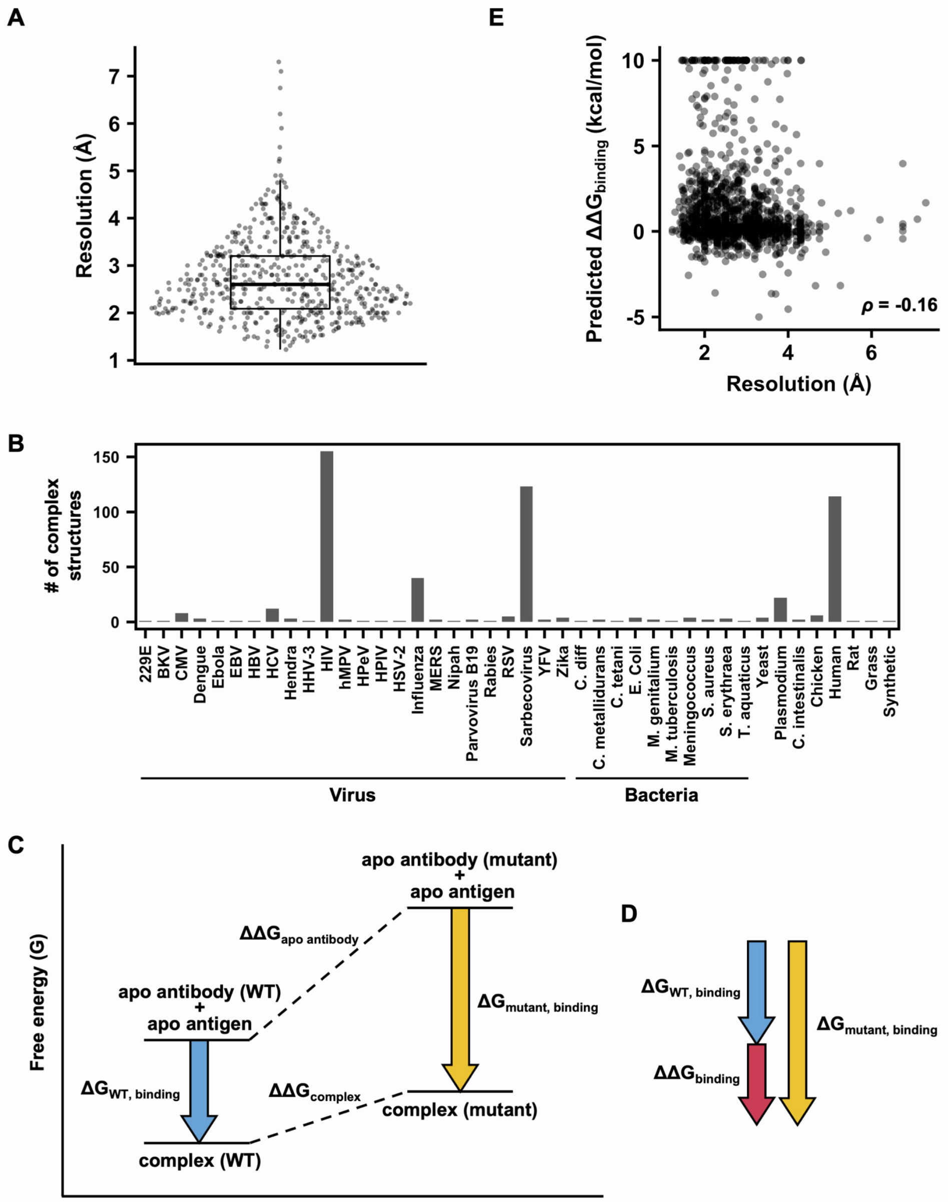

Figure S1. Overview of the dataset and ΔΔG calculation, Related to Figure 1. (A) Resolutions (in Å) of the 544 antibody-antigen complex structures are shown. **(B)** Paratope residues with allelic polymorphisms were identified in 544 antibody-antigen complex structures. The species of the antigen among these 544 antibody-antigen complex structures are plotted, with the occurrence frequency shown on the y-axis. **(C)** A free energy diagram of binding between antibody and antigen is shown. Upon mutation of the antibody, the free energy (G) of both the complex and the apo antibody may change, which can be quantified as $\Delta\Delta G_{complex}$ and $\Delta\Delta G_{apo}$

$_{antibody}$, respectively. Blue arrow indicates the ΔG of binding of the wild-type (WT) antibody ($\Delta G_{WT, binding}$), whereas the yellow arrow indicates the ΔG of binding of the mutant antibody ($\Delta G_{mutant, binding}$). **(D)** The difference between $\Delta G_{WT, binding}$ and $\Delta G_{mutant, binding}$ is represented by $\Delta\Delta G_{binding}$, which can be quantified as $\Delta\Delta G_{complex}$ – $\Delta\Delta G_{apo\ antibody}$. That is, $\Delta\Delta G_{binding} = \Delta G_{mutant, binding} - \Delta G_{WT, binding} = \Delta\Delta G_{complex} - \Delta\Delta G_{apo\ antibody}$. In this example here, the mutation strengthens the binding since it destabilizes the apo antibody to a greater extent than the complex. **(E)** The relationship between resolution and $\Delta\Delta G_{binding}$ of the 1,150 paratope allelic polymorphisms is shown. The Spearman's rank correlation coefficient (ρ) is indicated. Paratope allelic polymorphisms with predicted $\Delta\Delta G$ > 10 kcal/mol are shown as 10 kcal/mol. Paratope allelic polymorphisms with predicted $\Delta\Delta G$ < -5 kcal/mol are shown as -5 kcal/mol. (**A and E**) One data point represents one paratope allelic polymorphism.

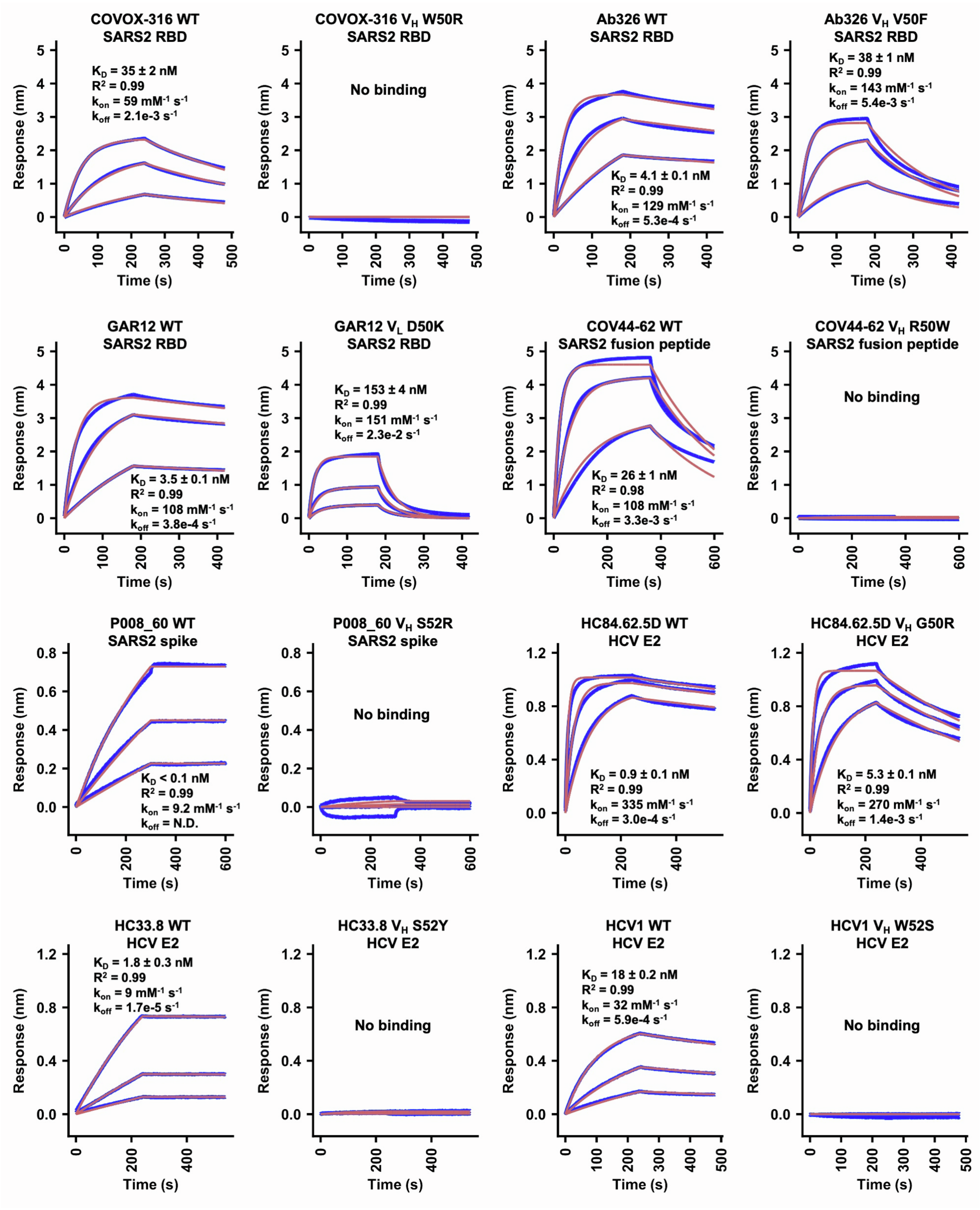
COVOX-316 WT
SARS2 RBD
K_D = 35 ± 2 nM
R² = 0.99
k_on = 59 mM⁻¹ s⁻¹
k_off = 2.1e-3 s⁻¹
COVOX-316 V_H W50R
SARS2 RBD
No binding
Ab326 WT
SARS2 RBD
K_D = 4.1 ± 0.1 nM
R² = 0.99
k_on = 129 mM⁻¹ s⁻¹
k_off = 5.3e-4 s⁻¹
Ab326 V_H V50F
SARS2 RBD
K_D = 38 ± 1 nM
R² = 0.99
k_on = 143 mM⁻¹ s⁻¹
k_off = 5.4e-3 s⁻¹
GAR12 WT
SARS2 RBD
K_D = 3.5 ± 0.1 nM
R² = 0.99
k_on = 108 mM⁻¹ s⁻¹
k_off = 3.8e-4 s⁻¹
GAR12 V_L D50K
SARS2 RBD
K_D = 153 ± 4 nM
R² = 0.99
k_on = 151 mM⁻¹ s⁻¹
k_off = 2.3e-2 s⁻¹
COV44-62 WT
SARS2 fusion peptide
K_D = 26 ± 1 nM
R² = 0.98
k_on = 108 mM⁻¹ s⁻¹
k_off = 3.3e-3 s⁻¹
COV44-62 V_H R50W
SARS2 fusion peptide
No binding
P008_60 WT
SARS2 spike
K_D < 0.1 nM
R² = 0.99
k_on = 9.2 mM⁻¹ s⁻¹
k_off = N.D.
P008_60 V_H S52R
SARS2 spike
No binding
HC84.62.5D WT
HCV E2
K_D = 0.9 ± 0.1 nM
R² = 0.99
k_on = 335 mM⁻¹ s⁻¹
k_off = 3.0e-4 s⁻¹
HC84.62.5D V_H G50R
HCV E2
K_D = 5.3 ± 0.1 nM
R² = 0.99
k_on = 270 mM⁻¹ s⁻¹
k_off = 1.4e-3 s⁻¹
HC33.8 WT
HCV E2
K_D = 1.8 ± 0.3 nM
R² = 0.99
k_on = 9 mM⁻¹ s⁻¹
k_off = 1.7e-5 s⁻¹
HC33.8 V_H S52Y
HCV E2
No binding
HCV1 WT
HCV E2
K_D = 18 ± 0.2 nM
R² = 0.99
k_on = 32 mM⁻¹ s⁻¹
k_off = 5.9e-4 s⁻¹
HCV1 V_H W52S
HCV E2
No binding
Response (nm)
Time (s)

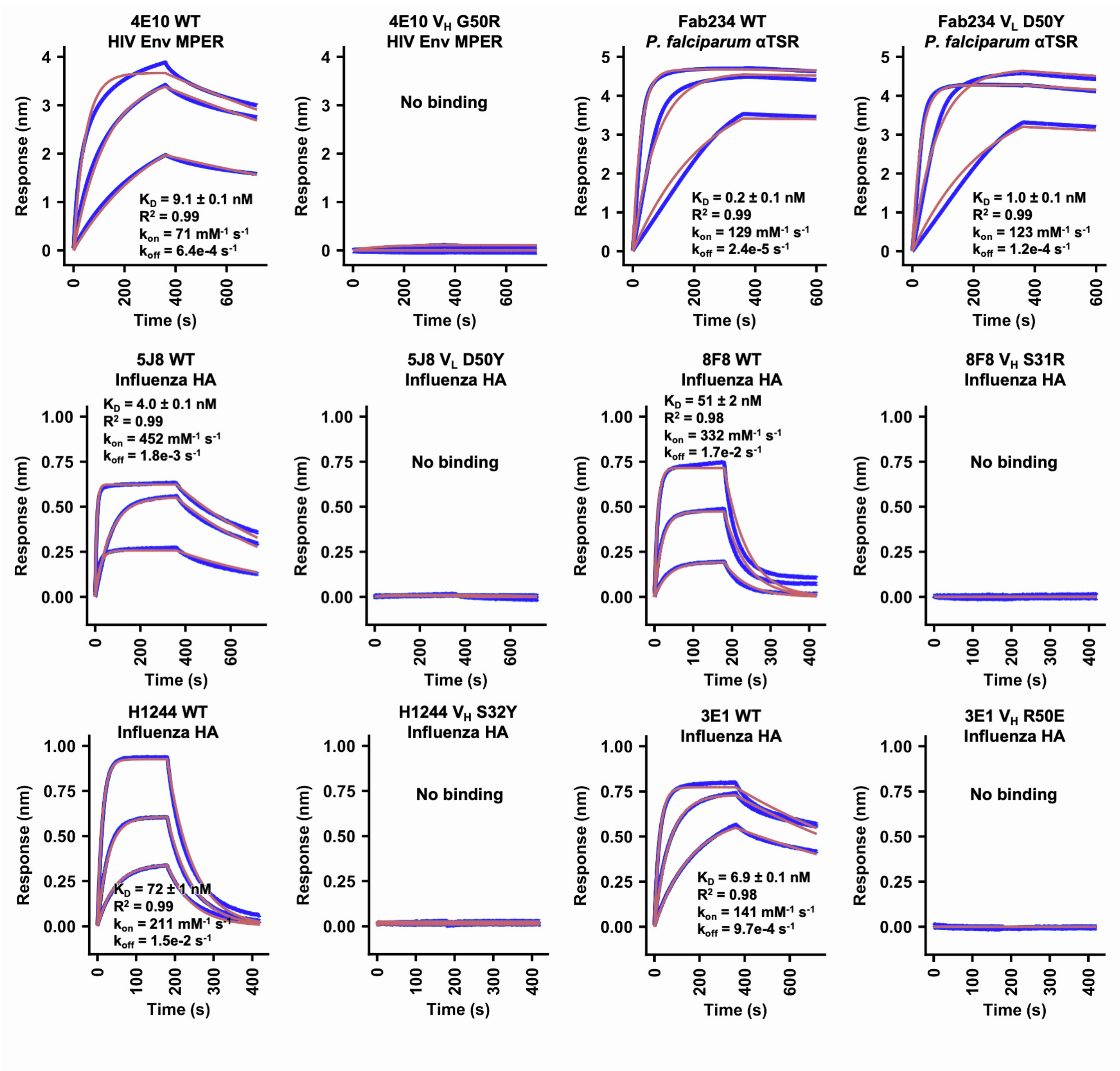

Figure S2. Sensorgrams for binding of Fabs to recombinantly expressed antigens, Related to Figures 3 and 4. Binding kinetics of different Fabs against their corresponding recombinantly expressed antigens were measured by biolayer interferometry (BLI). Y-axis represents the response. Blue lines represent the response curve and red lines represent a 1:1 binding model. Binding kinetics were measured for three Fab or antigen concentrations. Dissociation constant (K_D) and the goodness of model fitting (R^2) are indicated. Of note, in cases where binding of

mutated antibodies was detectable, the change in K_D could largely be attributed to the change in the k_{off}, rather than k_{on} values. N.D. indicates not detectable.

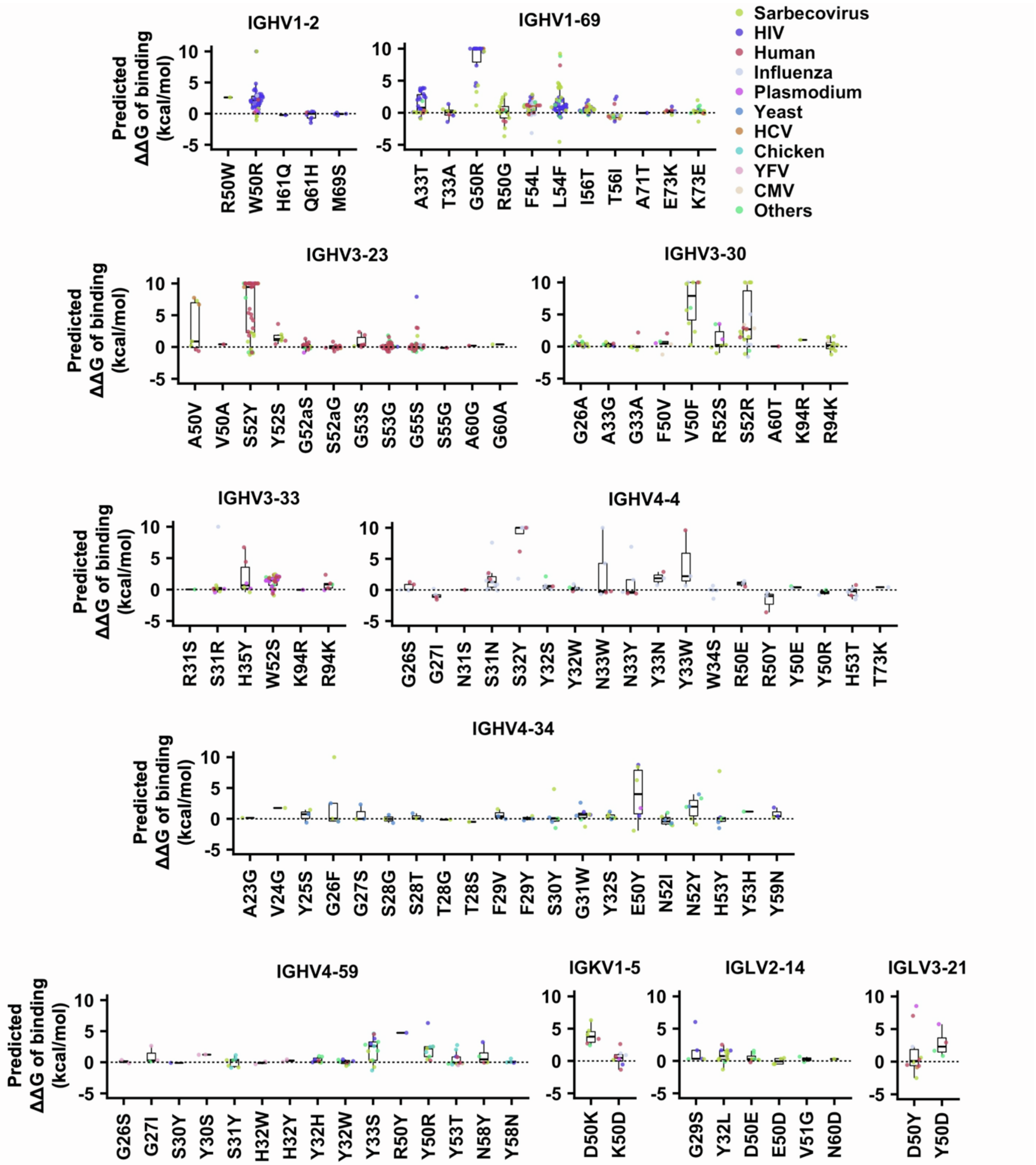

Figure S3. Distributions of predicted $\Delta\Delta G_{binding}$ of paratope allelic polymorphisms, Related to Figures 3 and 4. The distributions of predicted $\Delta\Delta G_{binding}$ of paratope allelic polymorphisms in antibodies encoded by the indicated V genes are shown. Paratope allelic polymorphisms are categorized by their identities and colored by the antigens. One data point represents one paratope allelic polymorphism. Paratope allelic polymorphisms with predicted $\Delta\Delta G$ > 10 kcal/mol

are shown as 10 kcal/mol. Paratope allelic polymorphisms with predicted $\Delta\Delta G < -5$ kcal/mol are shown as -5 kcal/mol.

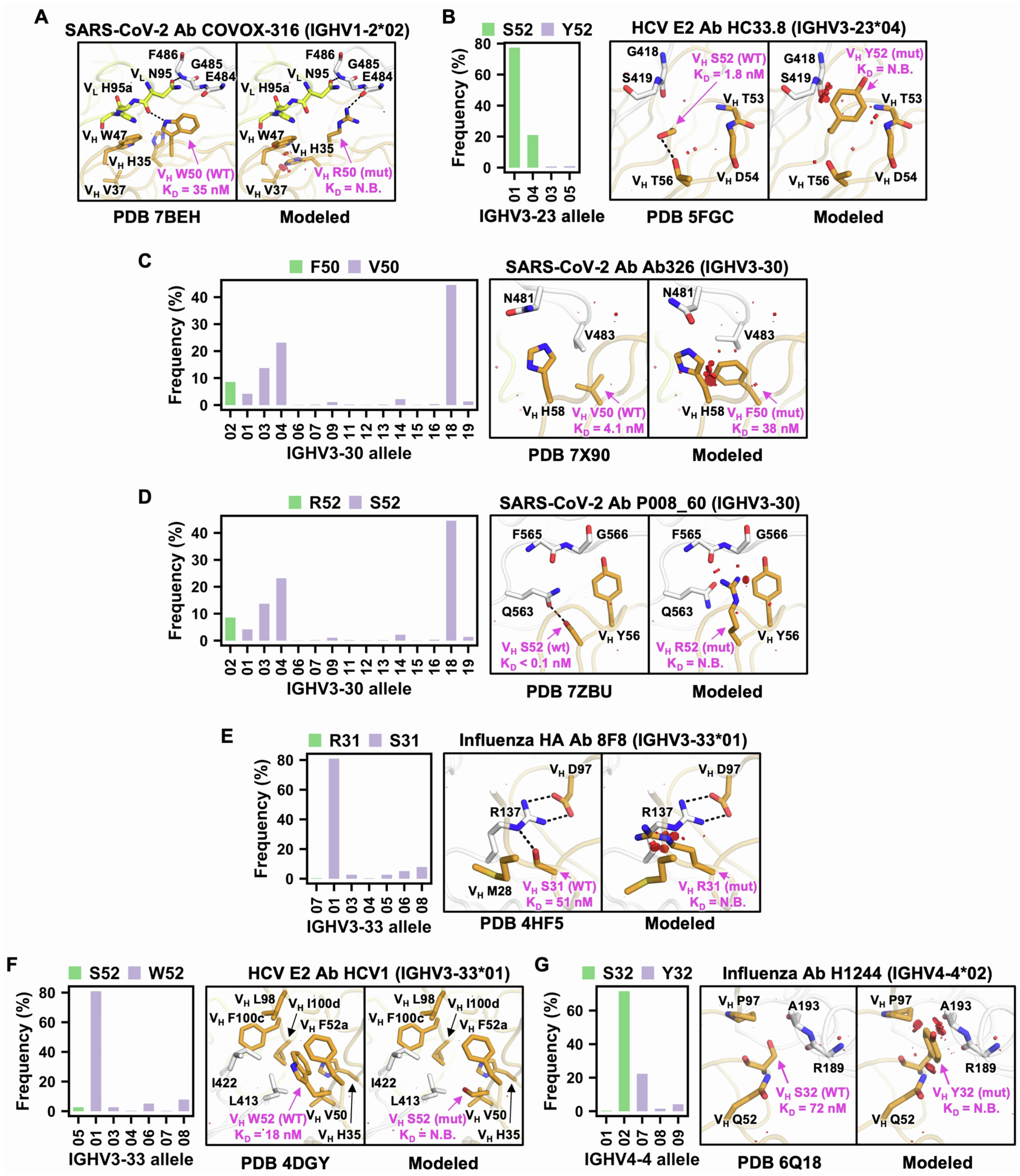

Figure S4. Structural analysis of selected paratope allelic polymorphisms, Related to Figures 3 and 4. The structural effects of paratope allelic polymorphisms **(A)** V_H W50R of antibody COVOX-316 in complex with the receptor binding domain (RBD) of SARS-CoV-2 spike (PDB 7BEH) [S1], **(B)** V_H S52Y of antibody H33.8 in complex with a peptide fragment of HCV E2

(PDB 5FGC) [S2], **(C)** V_H V50F of antibody Ab326 in complex with the RBD of SARS-CoV-2 spike (PDB 7X90) [S3], **(D)** V_H S52R of antibody P008_60 in complex with SARS-CoV-2 spike monomer (PDB 7ZBU) [S4], **(E)** V_H S31R of antibody 8F8 in complex with influenza H2N2 A/Japan/305/1957 hemagglutinin (PDB 4HF5) [S5], **(F)** V_H W52S of antibody HCV1 in complex with a peptide fragment of HCV E2 (PDB 4DGY) [S6], and **(G)** V_H S32Y of antibody H1244 in complex with influenza H1N1 A/Beijing/262/1995 hemagglutinin head domain (PDB 6Q18) [S7], are modeled by FoldX [S8]. The V gene and allele usage for each antibody are indicated. Of note, the allele usages for Ab326 and P008_60 cannot be assigned unambiguously. Binding dissociation constant (K_D) values of wild type and mutant to the antigens were measured by BLI and are indicated. Structure visualization was generated using the same style and format as **Figure 4**. The bar charts on the right in each panel shows the allele usages of different V genes. The y-axis of each bar chart represents the allele frequency among antibodies in GenBank that are encoded by the indicated V gene. Bar color represents the amino-acid identity at the indicated residue position.

Table S2. Summary of experimentally validated paratope allelic polymorphisms, Related to Figures 3 and 4.

PDB	Antibody	Antigen	Allelic polymophism	WT freq (%)[a]	Predicted $\Delta\Delta G_{binding}$ (kcal/mol)[b]	WT K_D (nM)	Mutant K_D (nM)
8DXT	GAR12	SARS-CoV-2 spike	IGKV1-5 D50K	16.3	6.3	3.5	153
7BEH	COVOX-316	SARS-CoV-2 spike	IGHV1-2 W50R	91.8	17.2	35	No binding
7X90	Ab326	SARS-CoV-2 spike	IGHV3-30 V50F	91.5	5.7	4.1	38
8D36	COV44-62	SARS-CoV-2 spike	IGHV1-2 R50W	8.2	2.6	26	No binding
7ZBU	P008_60	SARS-CoV-2 spike	IGHV3-30 S52R	91.5	11.7	<0.1	No binding
4Z0X	HC84.62.5D	HCV E2	IGHV1-69 G50R	83.6	14.2	0.9	5.3
5FGC	HC33.8	HCV E2	IGHV3-23 S52Y	98.4	20.6	1.8	No binding
4DGY	HCV1	HCV E2	IGHV3-33 W52S	97.3	2.4	18	No binding
4XBE	4E10	HIV Env	IGHV1-69 G50R	83.6	12.6	9.1	No binding
5GJS	3E1	Influenza HA	IGHV4-4 R50E	22.3	1.4	6.9	No binding
6Q18	H1244	Influenza HA	IGHV4-4 S32Y	72.0	22.2	72	No binding
4HF5	8F8	Influenza HA	IGHV3-33 S31R	99.7	17.9	51	No binding
4M5Z	5J8	Influenza HA	IGLV3-21 D50Y	66.3	2.3	4.0	No binding
7RXI	Fab234	*P. falciparum* CSP	IGLV3-21 D50Y	66.3	8.5	0.2	1.0

[a] Occurrence frequency of individual alleles of the indicated germline genes was estimated based on antibody sequences downloaded from GenBank (www.ncbi.nlm.nih.gov/genbank) [S9] (**see STAR Methods**). The occurrence frequency of alleles that encode the wild-type (WT) amino acid at the indicated position of the corresponding antibody is listed. For example, antibody GAR12 is encoded by IGKV1-5 with Asp50. Among all the IGKV1-5 antibodies in GenBank, 16.3% were assigned to IGKV1-5 alleles that encode Asp50 in the germline.

[b] $\Delta\Delta G_{binding}$ was predicted using FoldX [S8].

Supplemental References

S1. Dejnirattisai, W., Zhou, D., Ginn, H.M., Duyvesteyn, H.M.E., Supasa, P., Case, J.B., Zhao, Y., Walter, T.S., Mentzer, A.J., Liu, C., et al. (2021). The antigenic anatomy of SARS-CoV-2 receptor binding domain. Cell *184*, 2183-2200.e22. 10.1016/j.cell.2021.02.032.

S2. Keck, Z.Y., Girard-Blanc, C., Wang, W., Lau, P., Zuiani, A., Rey, F.A., Krey, T., Diamond, M.S., and Foung, S.K. (2016). Antibody response to hypervariable region 1 interferes with broadly neutralizing antibodies to hepatitis C virus. J Virol *90*, 3112-3122. 10.1128/JVI.02458-15.

S3. Takeshita, M., Fukuyama, H., Kamada, K., Matsumoto, T., Makino-Okamura, C., Uchikubo-Kamo, T., Tomabechi, Y., Hanada, K., Moriyama, S., Takahashi, Y., et al. (2022). Potent SARS-CoV-2 neutralizing antibodies with therapeutic effects in two animal models. iScience *25*, 105596. 10.1016/j.isci.2022.105596.

S4. Seow, J., Khan, H., Rosa, A., Calvaresi, V., Graham, C., Pickering, S., Pye, V.E., Cronin, N.B., Huettner, I., Malim, M.H., et al. (2022). A neutralizing epitope on the SD1 domain of SARS-CoV-2 spike targeted following infection and vaccination. Cell Rep *40*, 111276. 10.1016/j.celrep.2022.111276.

S5. Xu, R., Krause, J.C., McBride, R., Paulson, J.C., Crowe, J.E., Jr., and Wilson, I.A. (2013). A recurring motif for antibody recognition of the receptor-binding site of influenza hemagglutinin. Nat Struct Mol Biol *20*, 363-370. 10.1038/nsmb.2500.

S6. Kong, L., Giang, E., Robbins, J.B., Stanfield, R.L., Burton, D.R., Wilson, I.A., and Law, M. (2012). Structural basis of hepatitis C virus neutralization by broadly neutralizing antibody HCV1. Proc Natl Acad Sci U S A *109*, 9499-9504. 10.1073/pnas.1202924109.

S7. McCarthy, K.R., Raymond, D.D., Do, K.T., Schmidt, A.G., and Harrison, S.C. (2019). Affinity maturation in a human humoral response to influenza hemagglutinin. Proc Natl Acad Sci U S A *116*, 26745-26751. 10.1073/pnas.1915620116.

S8. Delgado, J., Radusky, L.G., Cianferoni, D., and Serrano, L. (2019). FoldX 5.0: working with RNA, small molecules and a new graphical interface. Bioinformatics *35*, 4168-4169. 10.1093/bioinformatics/btz184.

S9. Benson, D.A., Cavanaugh, M., Clark, K., Karsch-Mizrachi, I., Lipman, D.J., Ostell, J., and Sayers, E.W. (2013). GenBank. Nucleic Acids Res *41*, D36-42. 10.1093/nar/gks1195.
